# Rigorous optimization and validation of potent RNA CAR T cell therapy for the treatment of common epithelial cancers expressing folate receptor

**DOI:** 10.18632/oncotarget.5029

**Published:** 2015-09-02

**Authors:** Keith Schutsky, De-Gang Song, Rachel Lynn, Jenessa B. Smith, Mathilde Poussin, Mariangela Figini, Yangbing Zhao, Daniel J. Powell

**Affiliations:** ^1^ Ovarian Cancer Research Center, Department of Obstetrics and Gynecology, Perelman School of Medicine, University of Pennsylvania, PA 19104, Philadelphia; ^2^ Department of Experimental Oncology and Molecular Medicine, Istituto Nazionale dei Tumori, 20133, Milan, Italy; ^3^ Department of Pathology & Laboratory Medicine, Abramson Cancer Center, Perelman School of Medicine, University of Pennsylvania, PA 19104, Philadelphia

**Keywords:** folate receptor alpha, chimeric antigen receptor, adoptive immunotherapy, ovarian cancer, T cells

## Abstract

Using lentiviral technology, we recently demonstrated that incorporation of CD27 costimulation into CARs greatly improves antitumor activity and T cell persistence. Still, virus-mediated gene transfer is expensive, laborious and enables long-term persistence, creating therapies which cannot be easily discontinued if toxic. To address these concerns, we utilized a non-integrating RNA platform to engineer human T cells to express FRα-specific, CD27 CARs and tested their capacity to eliminate human FRα^+^ cancer. Novel CARs comprised of human components were constructed, C4-27z and C4opt-27z, a codon-optimized variant created for efficient expression. Following RNA electroporation, C4-27z and C4opt-27z CAR expression is initially ubiquitous but progressively declines across T cell populations. In addition, C4-27z and C4opt-27z RNA CAR T cells secrete high levels of Th-1 cytokines and display strong cytolytic function against human FRα^+^ cancers in a time- and antigen-dependent manner. Further, C4-27z and C4opt-27z CAR T cells exhibit significant proliferation *in vivo*, facilitate the complete regression of fully disseminated human ovarian cancer xenografts in mice and reduce the progression of solid ovarian cancer. These results advocate for rapid progression of C4opt-27z RNA CAR to the clinic and establish a new paradigm for preclinical optimization and validation of RNA CAR candidates destined for clinical translation.

## INTRODUCTION

Due to difficulties associated with genetically modifying primary T lymphocytes using non-viral based systems, investigators have generally utilized retroviral and lentiviral vectors in experiments that required high levels of transgene expression and viability in human T cells [1–3]. However, several factors may restrict the use of viral vectors in clinical application including the time and cost required for viral vector production, restrictions on the size and number of genes that can be packaged into vectors, risk of insertional mutagenesis and possible safety issues associated with immune-mediated toxicity stemming from long-term persistence and activity of engineered T cells [3, 4]. Adverse effects in patients related to administration of genetically-redirected T cells, including cytokine storm, cardiac arrhythmia, respiratory failure, seizures and even mortality have been reported using viral-based CAR T cell therapy [5–11]. In light of these potential events, a need exists for alternative, safe and effective gene transfer in T lymphocyte research, CAR development and clinical application.

Traditionally, gene transfection of T lymphocytes *ex vivo* has been problematic, often hindered by low transfection efficiency and irreversible toxicity caused by transfection agents acting on primary cell types, including T cells [12–15]. Although T lymphocytes are refractory to most kinds of nonviral gene delivery, RNA electroporation is emerging as a particularly useful strategy to introduce a gene of interest into T lymphocytes, and the concept of utilizing RNA therapeutically has received considerable attention during the past decade [3, 16]. Recently, it was reported that electroporation with RNA could be utilized to obtain high levels of CAR-T cell gene transfer efficiency and low electroporation-related apoptosis [3]. Furthermore, the transmission of CAR-based RNAs into T lymphocytes redirected these lymphocytes to recognize and destroy human leukemia *in vivo*, using various mouse models [17–21].

Hence, expression of CARs using a RNA platform represents an alternative method of potentially testing CARs clinically (with additional safety) where there may be concerns about possible cumulative “on-target, off-tumor” toxic effects, as the metabolism of RNA over time ensures complete removal of the CAR in the patient without relying on suicide induction systems. RNA CAR expression is self-limiting, serving as its own vehicle for toxicity attenuation. In contrast to longer-term, integrating viral expression systems, RNA transfection may permit rapid iterative changes in CAR design and the possibility of moving toward a good manufacturing practice (GMP)-compliant system with potentially lower expenses and less complicated pharmacokinetic testing as opposed to lentiviral or retroviral vectors [17, 19]. The RNA CAR approach may be limited, however, in situations where long-term CAR expression is required for complete response (i.e. bulky, aggressive solid tumor malignancies) or when repeated T cell infusion is not possible [22].

Long-term CAR T cell persistence and increased T cell activity can be achieved by addition of costimulatory domains to the intracellular portion of the CAR construct. Costimulatory endodomains from CD28, CD134 (OX-40) and CD137 (4-1BB) in CARs significantly enhances the ability of CAR T cells to secrete cytokines, proliferate, persist and produce antitumor effects in rodents [9–11, 23–28]. Similarly, CAR therapy utilizing one or more costimulatory molecule domains (2^nd^ and 3^rd^ generation CARs) have shown considerable benefit in patients with lymphoma and other cancers [29, 30]. Recently, we demonstrated that the costimulatory molecule, CD27, actively enhances CAR T cell function, expansion and survival *in vivo* [28, 31]. Human T cells virally transduced to express a folate receptor-α (FRα)-specific CAR, comprised of an extracellular murine anti-human FRα MOv-19 scFv and an intracellular CD3 zeta (CD3ζ) chain signaling module in tandem with a CD27 costimulatory endodomain displayed enhanced cytokine release, cytolytic function and proliferation *in vivo*, thereby rationalizing the incorporation of CD27 costimulation in CAR-T therapy for FRα+ cancer.

Elevated expression of the glycosylphosphatidylinositol-anchored protein, FRα, occurs in many human malignancies, including ovary, breast, kidney, lung, colon, rectum, head, neck and brain cancers [32–38], and is limited in normal tissues [39]. Recently, it was suggested that the degree of FRα content in a tumor may be utilized for prognostic purposes, with higher expression being especially deleterious [32]. Notably, the number of human tumors reported to express FRα is rapidly growing [39]. Therefore, CAR-T cell therapies specifically targeting FRαmay be of considerable benefit in the clinic. While 90% of ovarian cancers (OC) over-express FRα, the vast majority of somatic tissues do not, making this cancer type particularly relevant when examining the efficacy of FRα CAR T cell therapies and their potential for immune toxicity [33, 39]. Moreover, OC ranks as the fifth leading cause of cancer mortality among women, with one in 70 females developing OC in her lifetime [40]. Currently, there are no diagnostics for early detection, and the majority of these cancers are not diagnosed until the cancer has metastasized, making the disease extremely difficult to treat. Hence, CAR-T cell based therapies that effectively combat OC, when widely disseminated, would be extremely beneficial. Although viral CAR-T cell therapies have been shown to effectively combat OC in preclinical models, there are no reports of clinically effective RNA CAR-T cell therapies in OC [28, 41–43].

To bring safe, effective therapy to OC patients, we utilized an RNA platform to create and test two novel FRα-specific CARs, C4-27z and C4opt-27z, which, when electroporated as RNA into primary human T lymphocytes, lead to efficient, but transient, CAR expression. Unlike, past FRα-specific CARs [31, 44, 45], these CARs are fully human in composition with reduced risk for inducing transgene immunogenicity and anaphylaxis which can accompany use of CARs bearing murine components [44, 46]. Furthermore, the C4-opt-27z CAR is codon optimized for efficient expression in human T cells. Importantly, robust surface expression of these CARs on T cells leads to vigorous Th-1 cytokine secretion and potent cytolytic activity against a number of FRα-expressing ovarian cancer lines. Further, multiple injections of T lymphocytes expressing C4-27z or C4opt-27z CAR provide complete remission against fully disseminated human ovarian tumor xenografts in rodents, which correlates with significant CD4^+^ and CD8^+^ T cell expansion in peripheral blood. C4-27z and C4opt-27z RNA CAR therapy also significantly reduce the progression of solid ovarian cancer *in vivo*. Notably, codon optimization of the CAR modestly, but reproducibly, improved cancer cell killing *in vitro* and *in vivo* under suboptimal treatment dosing schedule, making it a strong candidate for use in clinical application in patients with FRα-expressing cancers.

## RESULTS

### CAR construction

**Figure 1:**
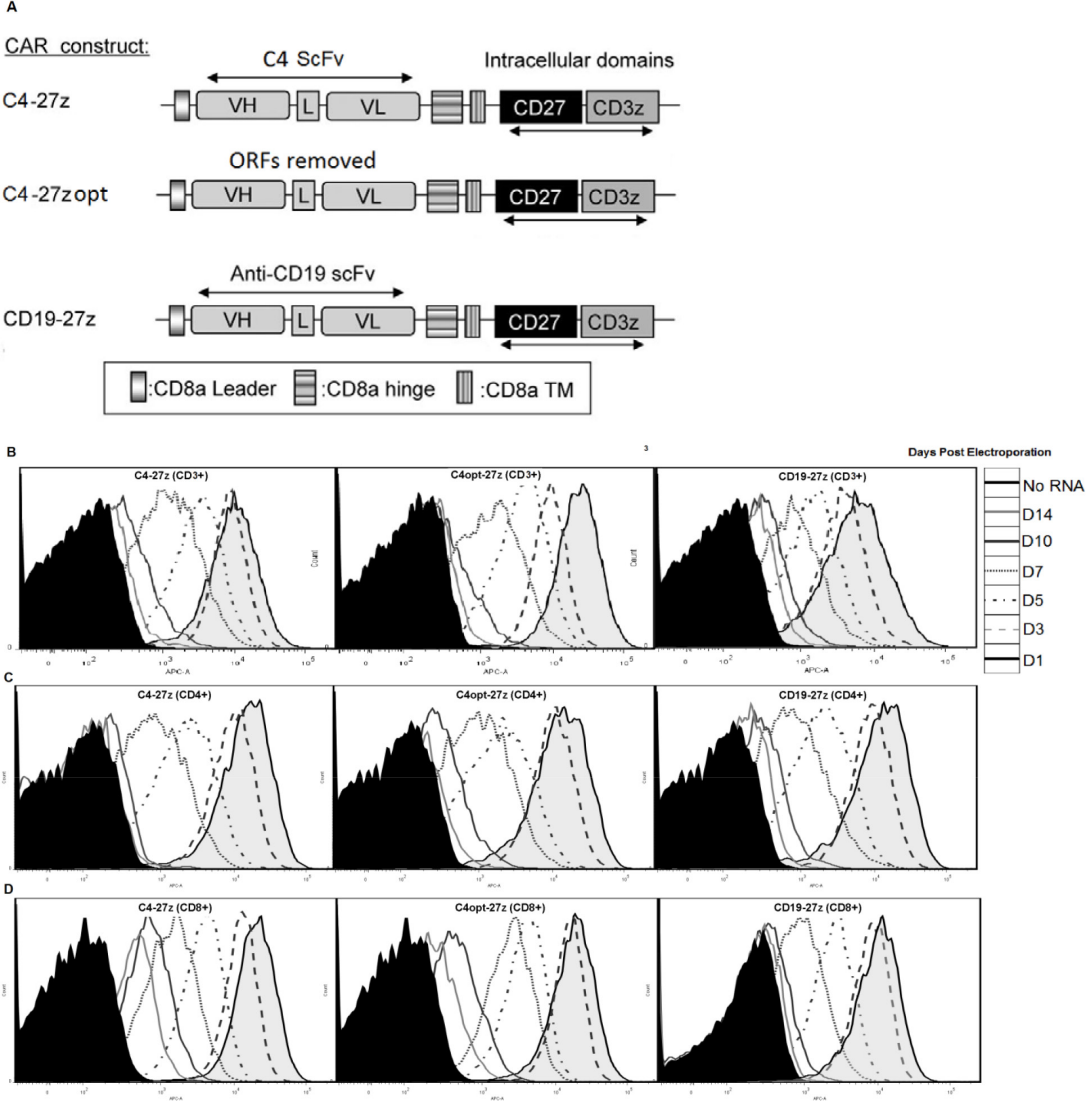

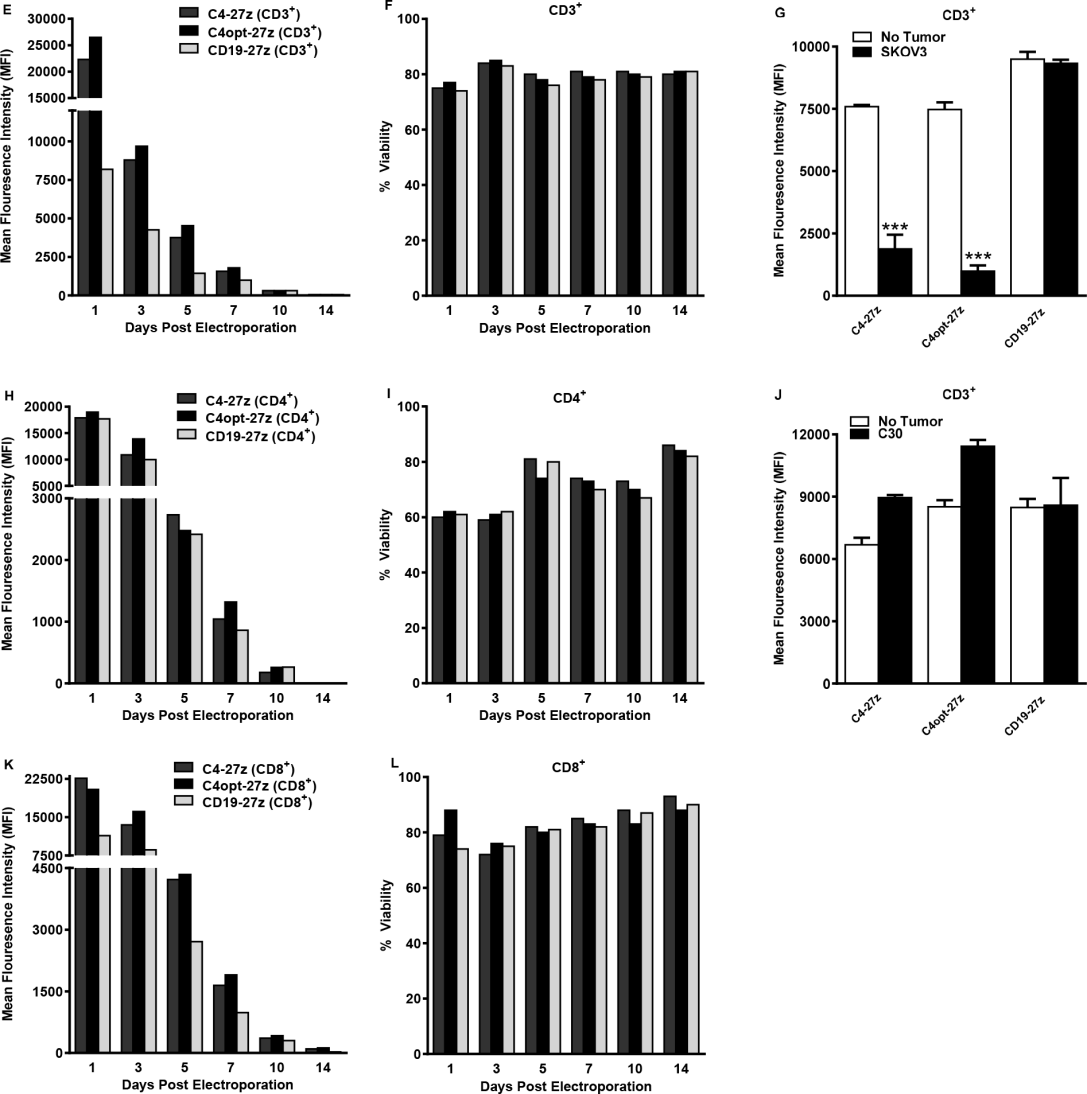
Generation, expression and viability of FRα-specific CAR-transfected human T lymphocytes *in vitro* **A.** A schematic representation of FRα-specific, C4 or CD19 (control) CAR constructs containing the CD3ζ cytolytic domain in combination with the CD27 costimulatory molecule. VL, variable light chain; L, linker; VH, variable heavy chain; TM, transmembrane region. **B–D, E, H, K.** CAR expression as measured by APC-A or change in mean fluorescence intensity (MFI) at different time points after electroporation with C4-27z, C4opt-27z or CD19–27z RNA. Electroporated CD3^+^, CD4^+^ or CD8^+^ T cells (containing no RNA) were used as negative controls (filled histograms). **F, I, L.** Cell viability was detected using 7-AAD (BD Viaprobe). **G, H.** Expression of C4-27z and C4opt-27z, but not CD19–27z (control CAR) declines rapidly (measured at 72 hr) after 24 hr coincubation with FRα^+^ SKOV3 tumor on day 1 (student *t* test, *p* < .001). Conversely, expression of C4-27z, C4opt-27z and CD19–27z do not change after co-incubation with FRα^−^ C30.

FRα-specific CARs containing the fully human scFV C4, which has specificity for FRα [47], were constructed. FRα constructs were composed of the C4 scFv linked to a CD8α hinge and transmembrane region, followed by a CD3ζ signaling moiety in tandem with the CD27 intracellular signaling motif (C4-27z, Figure 1A). To increase the efficiency of CAR expression and address the potential for off-frame transcription, codons were optimized and all internal open reading frames (ORFs) were removed with one exception, creating the C4opt-27z CAR. A single ORF in the reverse complement strand at nucleotide position 1511 could not be removed as a switch from CAC to CAT (His at amino acid position 493) which would have created a new ORF in the antisense strand. Fortunately, a stop codon starting at position 1496 ensured that this internal ORF would only yield a five amino acids peptide (H-L-A-D-Y), if ever translated, too small to produce an immunologically functional protein. A CD19-specific CAR containing CD3ζ and CD27 signaling motifs (CD19–27z) was constructed to control for antigen specificity. CAR constructs were subcloned into a pD-A.lenti cloning site.2bg.150A vector (PDA) that was optimized for T cell transfection, CAR expression and RNA production [18]. Transgene expression was driven by the T7 promoter.

### RNA electroporation of human T cells results in high CAR expression efficiency and viability

RNA gene transfer technology established for clinical application was used, as previously described [19, 48]. The RNA-based, PDA vector was utilized to transfect human T cells which then efficiently expressed anti-FRα or anti-CD19 CARs (Figure 1B–1D). Strikingly, transfection efficiency for C4-27z, C4opt-27z and CD19-27z CAR T cells approached 100% during the first 24 hr, declining at a rate similar in CD3^+^, CD4^+^ and CD8^+^ T cell populations (Figures 1B–1D, 1E, 1H, 1K). Reproducibly, electroporation with C4opt-27z RNA resulted in higher levels of surface CAR expression than with the parental C4-27z, indicating enhanced translation and expression. Importantly, transgene surface expression was detectable up to 10 days after RNA electroporation in C4-27z, C4opt-27z and CD19-27z CAR T cells. Viability for CD3^+^, CD4^+^ and CD8^+^ T cells after CAR electroporation was ∼65–80% during the course of experiments, indicating that neither electroporation nor transgene expression caused irreversible damage or significantly affected T cell health (Figures 1F, 1I, 1L). CAR T cell expression, viability and the rate of CAR expression loss were dependent on the concentration of input RNA (Supplemental Figures 1A–1I). In general, higher concentrations of RNA produced greater CAR T cell expression for longer periods at the expense of viability during the first 24–72 hours following electroporation, although T cells recovered in media containing 50–100 IU/mL IL-2, regardless of RNA concentration. Notably, after 24 hours of coincubation with FRα-expressing cancer cells (SKOV3), C4-27z and C4opt-27z, but not CD19-27z, CAR expression declined rapidly in CD3^+^, CD4^+^ or CD8^+^ T cells, consistent with T cell activation and metabolism resulting in a greater loss of RNA-transcribed CAR expression (Figures 1G, Supplemental Figures 2A–2B). Conversely, CAR expression of C4-27z and C4opt-27z T cells was not affected after co-incubation with FRα^−^ C30 cancer cells (Figure 1J), suggesting that CAR activation is antigen (FRα)-specific.

### C4 and C4opt CAR T cells with CD27z signaling exert antigen-specific reactivity *in vitro*

**Figure 2:**
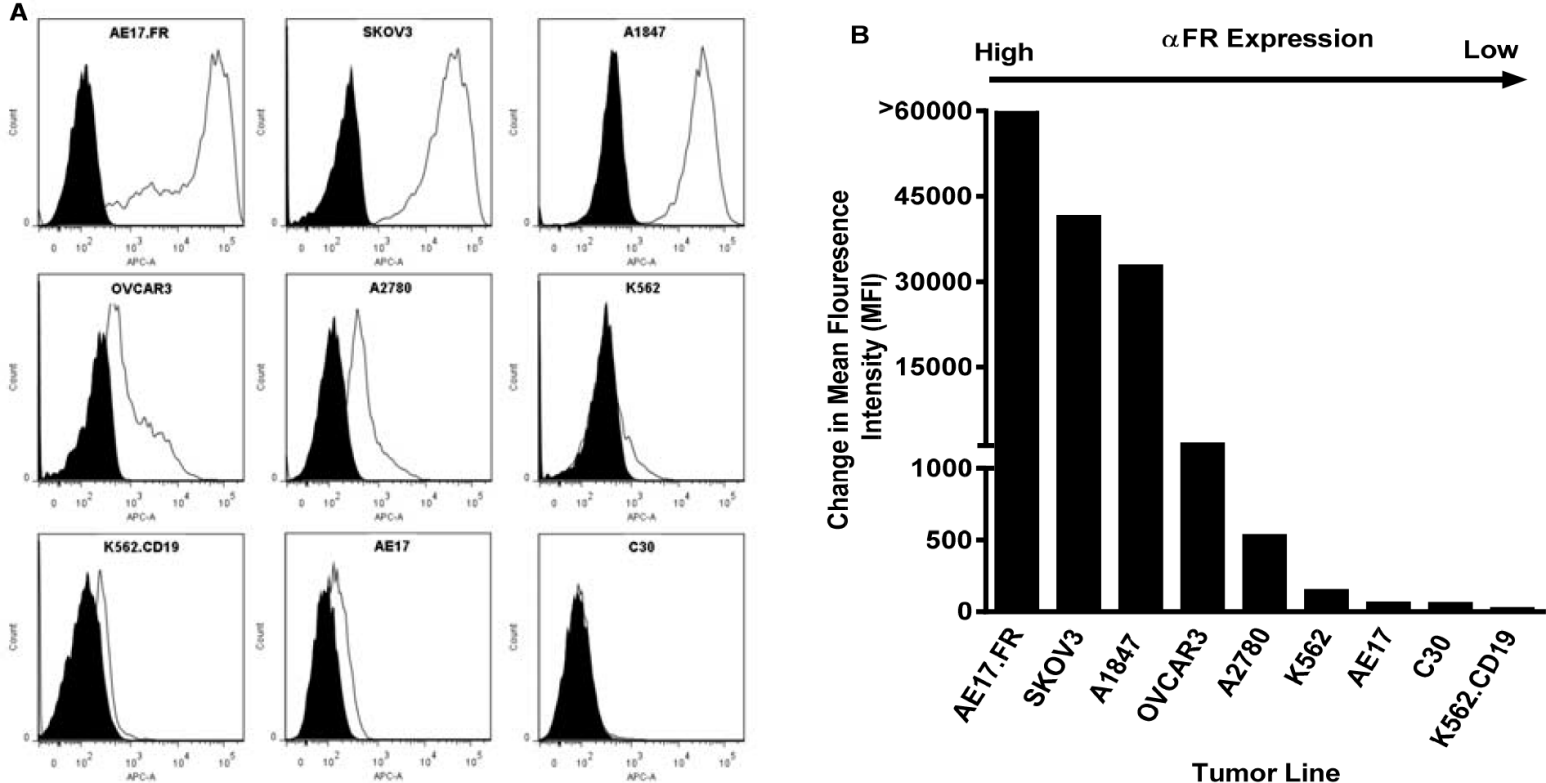
FRα surface expression of human ovarian cancer cell lines by flow cytometry **A.** FRα-specific mAb MOv18 was used to measure FRα expression of tumor cell lines by flow cytometry (open histogram), compared with IgG1 isotype controls (filled grey histogram). **B.** FRα expression as measured by the change in mean fluorescence intensity (MFI).

Ninety percent of human ovarian cancers express FRα, making such cancers particularly useful to examine functional activity of FRα-specific CAR T lymphocytes [33, 39]. Thus, a panel of established human ovarian cancer lines that express surface FRα at various levels were selected for immune assays (Figures 2A–2B). Flow cytometry results confirmed that ovarian cancer lines SKOV3, A1847, OVCAR3 and A2780 expressed surface FRα protein, as did a control line AE17.FRα (a mouse mesothelioma line engineered to express human FRα). K562.CD19 (a human erythomyeloblastoid leukemia cell line engineered to express human CD19) served as a control for CD19-27z CAR functions. C30, parental AE17, K562 and engineered K562.CD19 lines were mostly negative for FRα.

**Figure 3:**
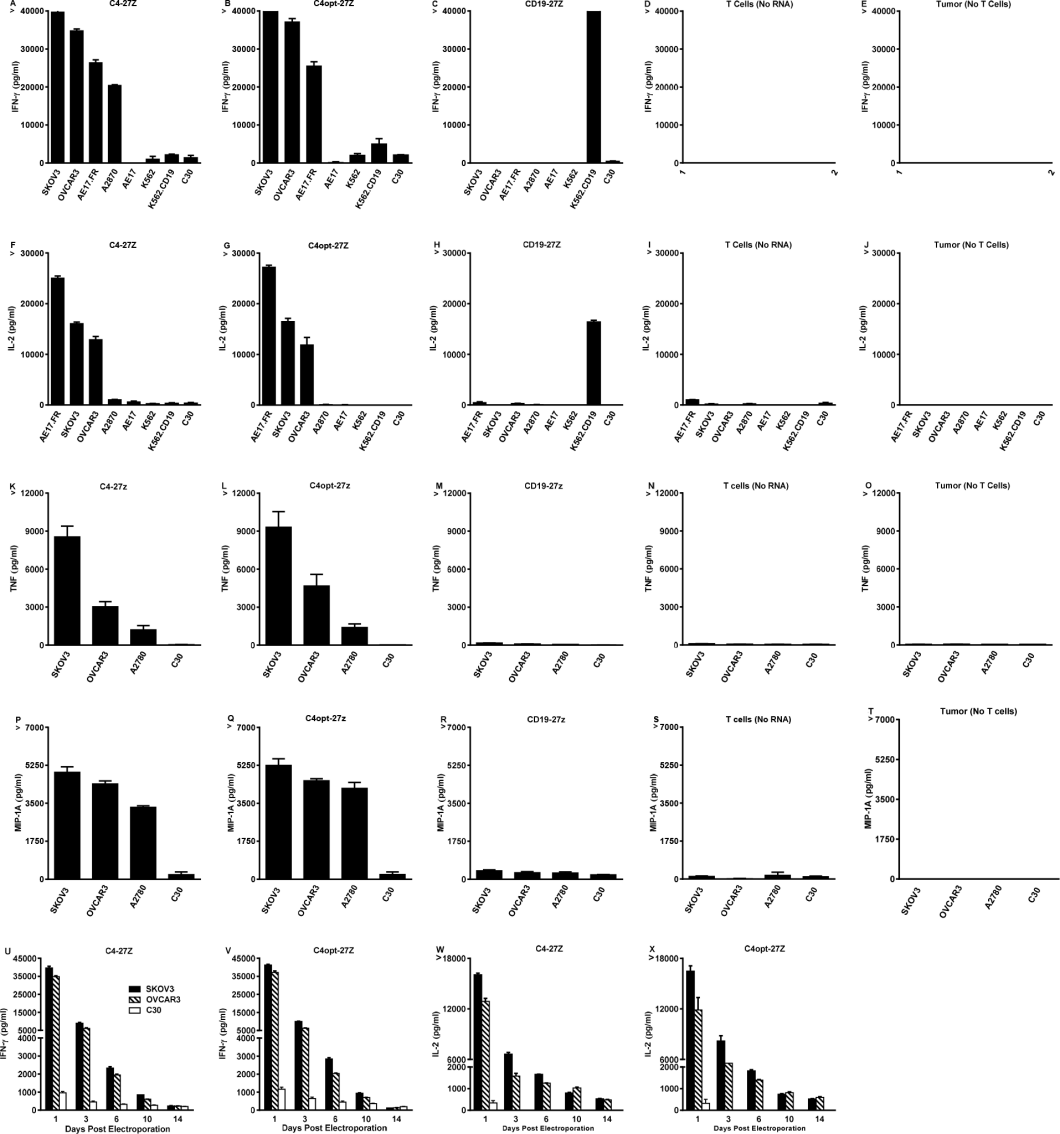
High levels of Th-1 cytokines are secreted by electroporated C4-27z and C4opt-27z RNA CAR T cells in response to FRα^+^ tumor cells **A–B, D–E.** Antigen-specific IFN-γ & IL-2 cytokine production by FRα-specific RNA CAR-transfected T cells ∼ 24 hr after coculture with the indicated tumor lines at a 1:1 ratio, measured with Elisa. **C, F.** CD19-27z CAR-T cells were used as negative controls. **G–L.** TNF-α and MIP-1A production was measured by human cytometric bead array (CBA) according to manufacturer's instructions. Non-electroporated T cells, electroporated T cells (containing no RNA) and tumor lines themselves did not produce appreciable levels of any cytokine (data not shown). **M–P.** Release of IFN-g & IL-2 declines with time and is dependent on the interval between RNA CAR electroporation and coincubation. Results are expressed as a mean +/− SEM of triplicate wells from 1 of at least 2 separate experiments.

To evaluate the extent of C4-27z and C4opt-27z antigen-specific reactivity *in vitro*, CAR RNA electroporated T cells and cancer cells were cocultured overnight and T cell reactivity was measured by proinflammatory cytokine secretion. C4-27z and C4opt-27z CAR T cells exclusively recognized FRα^+^ ovarian cancer lines, secreting high levels of Th-1 cytokines, including IFN-γ, IL-2, TNF-α and MIP-1A (Figures 3A–3B, 3F–3G, 3K–3L, 3P–3Q). Release of these Th-1 cytokines was FRαand CAR specific, as C4-27z and C4opt-27z CAR T cells produced little or undetectable levels of cytokines when stimulated by FRα^−^ cell lines, including C30, AE17, K562, and K562.CD19. C4-27z and C4opt-27z CAR T lymphocytes secreted low, but reliably detectable levels of Th-2 cytokines, including IL-4 and IL-10, in response to FRα^+^ cancer cells (Supplemental Figures 3A–3B, 3D–3E) although the response was considerably Th-1 biased. As expected, CD19-27z CAR T cells did not produce appreciable levels of cytokines after coculture, except when coincubated with K562.CD19 cells (Figures 3C, 3H, 3M, 3R). In addition, non-electroporated T cells, mock RNA electroporated T cells (containing no RNA), and cancer lines themselves did not secrete appreciable levels of cytokines (Figures 3D, 3I, 3N, 3S), illustrating a requirement for antigen-specificity in CAR T cell reactivity. Notably, levels of cytokine production exhibited by C4opt-27z RNA CAR T cells were dependent on the concentration of RNA used for electroporation, reaching a maximum at 5 μg RNA (Supplemental Figure 4A). In addition, non-specific cytokine secretion by CD19–27z CAR T cells increased at successively higher RNA concentrations in parallel cocultures (Supplemental Figure 4A). Interestingly, C4opt-27z and CD19–27z CAR T cells electroporated with escalating doses of RNA and then cocultured overnight with FRα^−^ C30 tumor displayed dose-dependent (but antigen-independent) CAR reactivity (Supplemental Figure 4B). Together, these results suggest that lower RNA concentrations (∼5 μg) may be used to obtain nearly optimal FR-specific effects while minimizing the baseline of non-specific immune activation.

Importantly, optimization of C4-27z did not negatively impact function, as C4opt-27z CAR T cells were equally, and often more, immunoreactive than C4-27z CAR T cells, which is consistent with equivalent, if not higher, levels of C4opt scFv CAR expression on the cell surface, compared to C4 CAR (Figures 1B–1D, 1E, 1H, 1K). In addition, CD4^+^ and CD8^+^ T cells that were isolated, expanded and electroporated separately with C4-27z or C4opt-27z CARs displayed robust reactivity, with each population producing Th-1 type cytokines in a FRα-dependent manner, and each population exhibiting a Th-1 response (Supplemental Figures 5A–5B, 5D–5E, 5G–5H, 5J–5K). Generally, CD4^+^ CAR T cells preferentially secreted IL-2 whereas CD8^+^ CAR T cells produced more IFN-γ. However, both T cell populations were able to produce IL-2 and IFN-γ in relatively high amounts although at levels less than total CD3^+^ CAR T cells, suggesting synergy between CD4^+^ and CD8^+^ CAR T cells in mediating the proinflammatory response. Finally, we tested if cytokine production by FRα-specific CAR T cells declined in a manner consistent with CAR expression level. CAR T cells, at progressively later times after RNA electroporation when CAR expression declines, were cocultured with FRα^+^ tumor lines overnight. Indeed, C4-27z and C4opt-27z RNA CAR T cells progressively secreted significantly lower levels of cytokines 3, 6 and 10 days post-electroporation and did not produce elevated levels of IFN-γ or IL-2 at day 14 when CAR expression was expired (Figures 3U–3X and 1B–1D).

### C4-27z, C4opt-27z CAR T cells display potent cytolytic function *in vitro*

**Figure 4:**
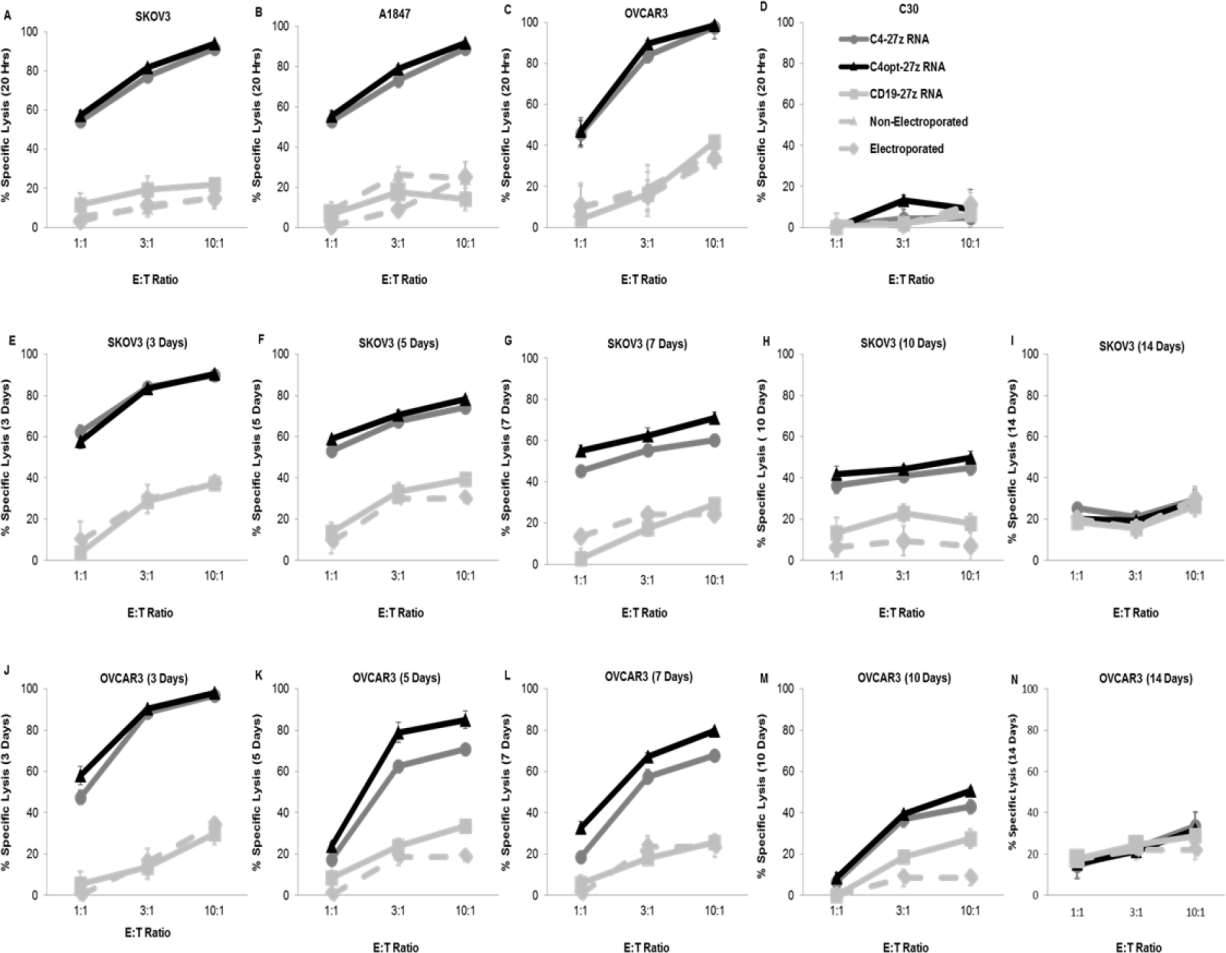
CAR-transfected, FRα-specific T cells show effective, time-dependent lytic function in a bioluminescent killing assay **A–D.** 24 hr after electroporation, C4-27z and C4opt-27z CAR T cells potently and specifically lyse FRα^+^ SKOV3, A1847 and OVCAR3 but do not kill FRα^−^ C30 cells at the indicated E/T ratios after coincubation with respective tumor cells. CD19-27z, electroporated T cells (containing no RNA) and non-electroporated T cells serve as negative controls. **E–N.** Killing potential of C4-27z and C4opt-27z RNA CAR T lymphocytes progressively declines during the 3, 5, 7, 10 or 14 d interval between RNA CAR electroporation and when cytolytic function in assessed. For all cytolytic assays, T cells are coincubated with tumor cells for ∼ 20 hr. Mean and SEM of six wells per data point is depicted.

Next, we assessed the FRα-specific cytolytic potential of RNA electroporated C4-27z, C4opt-27z and CD19-27z CAR T cells *in vitro*. RNA CAR T cells were cocultured overnight with FRα^+^ SKOV3, A1847, OVCAR3 or FRα^−^ C30 cancer cells expressing firefly luciferase and then assessed for bioluminescence. Luminescence results displayed in Figures 4A–4D reveal that C4-27z and C4opt-27z CAR T cells specifically destroyed FRα^+^ SKOV3, A1847 and OVCAR3 but not FRα^−^ C30 cells. Moreover, non-electroporated T cells, mock RNA electroporated T cells and CD19-27z CAR T cells did not appreciably lyse any of the control cancer lines tested. In general, C4opt-27z CAR T cells showed enhanced or comparable cytolytic potential compared to C4-27z CAR T cells. Interestingly, FRα-specific lytic function of C4opt-27z CAR T lymphocytes was dependent on the concentration of RNA utilized for electroporation (Supplemental Figure 6A). In addition, non-specific lysis by CD19-27z CAR T cells increased at successively higher RNA concentrations (Supplemental Figure 6B), mirroring cytokine release. C4opt-27z and CD19-27z CAR T cells also displayed elevated levels of antigen-independent lysis when electroporated at high RNA concentrations and then cocultured with FRα^−^ C30 cells (Supplemental Figures 6C–6D). Hence, low to moderate RNA concentrations may discriminate between FRα-specific effects and non-specific activation of CAR T cells themselves due to RNA electroporation. CD8^+^ C4-27z and C4opt-27z CAR T cells display greater cytolytic function than CD4^+^ C4 CAR T cells, particularly at high E/T ratios (Supplemental Figures 7A–7B), and similar levels of non-specific lysis (Supplemental Figures 7C–7D). Finally, we examined the cytolytic potential of C4-27z and C4opt-27z CAR T cells as a function of time. Similar to the rate of decline in CAR expression and cytokine production (Figures 1B–1D, 1E, 1H, 1K, 3U–3X), C4-27z and C4opt-27z CAR T cell killing potential decreased in a time-dependent fashion, returning to baseline levels 14 days after electroporation (Figure 4E–4N).

### C4-27z, C4opt-27z CAR T cells exhibit proliferation and potent antitumor function *in vivo*

**Figure 5:**
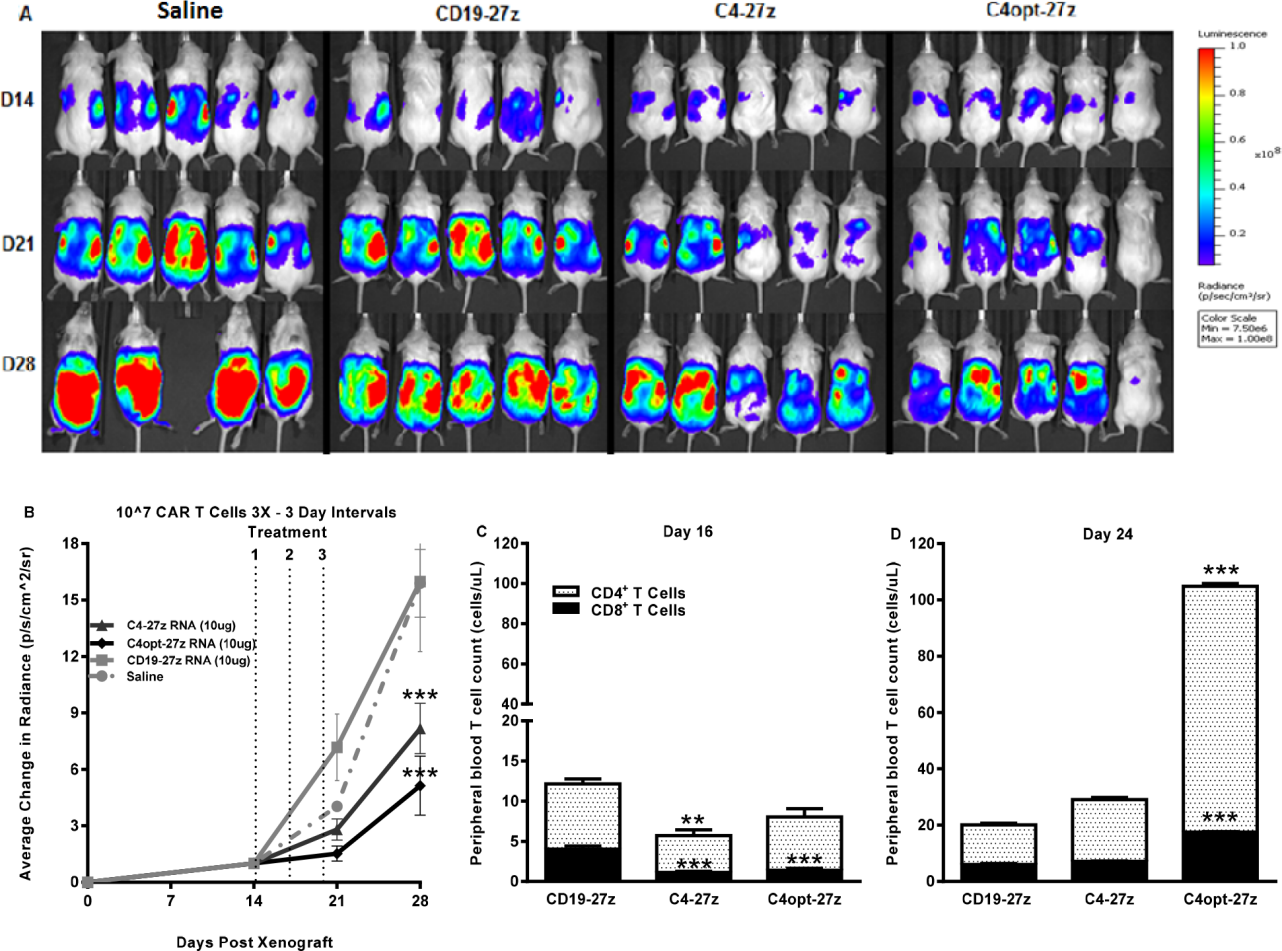
Human C4-27z and C4opt-27z RNA CAR T cells reduce the progression of pre-established tumors *in vivo* **A.** NSG mice bearing disseminated SKOV3 tumor were treated with intraperintoneal injections of 10^7^ CAR^+^ T cells on days 14, 17 and 20 and imaged weekly. **B.** Tumor growth was assessed by SKOV3 fLUC^+^ bioluminescence, which revealed reduced tumor progression in animals receiving FRα-specific C4-27z or C4opt-27z CAR T cell therapy (2-way ANOVA, *p* < .001). **C.** CD4^+^ and CD8^+^ C4-27z and C4opt-27z CAR T cells were largely absent from peripheral circulation in comparison to CD19-27z CAR T cells, suggesting FRα-specific migration to tumor sites (student *t* test, *p* < .01 – *p* < .001). **D.** Repeat administration of C4opt-27z CAR T cells resulted in significant expansion of CD4^+^ and CD8^+^ T cells in peripheral blood, which correlated with therapeutic efficacy of the C4opt-27z CAR (*p* < .001). CD4^+^ and CD8^+^ cells were quantitated from blood using the TruCount method. Mean cell concentration (cells/μl) +/− SEM for all mice in each treatment group is shown.

**Figure 6:**
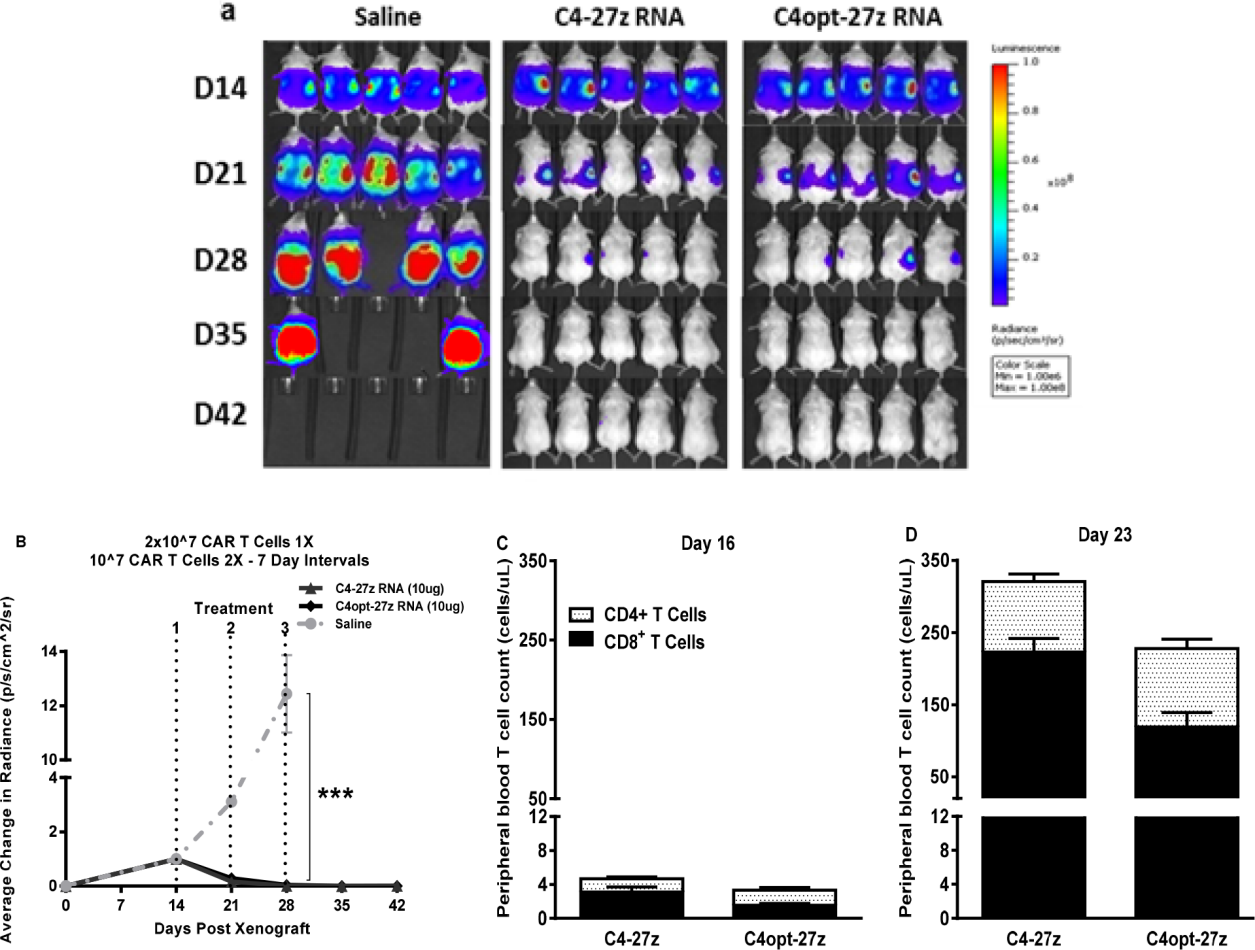
C4-27z and C4opt-27z RNA CAR T cells completely eliminate widely disseminated tumors *in vivo* **A.** NSG mice bearing disseminated SKOV3 tumor were treated with one intraperintoneal injection of 2 × 10^7^ CAR^+^ T cells on day 14 (loading dose) followed by a lower maintenance dose of 10^7^ CAR^+^ T cells on days 21 and 28. **B.** Tumor growth was assessed by SKOV3 fLUC^+^ bioluminescence, which demonstrates complete tumor remission in all C4-27z and C4opt-27z CAR T lymphocyte treated, tumor-bearing animals (2-way ANOVA, *p* < .001). **C.** CD4^+^ and CD8^+^ C4-27z and C4opt-27z CAR T cells were initially present at low numbers in peripheral circulation, suggesting FRα-specific CAR T cell migration to specific tumor locales. **D–E.** Conversely, successive administration of C4-27z and C4opt-27z CAR T cells resulted in extensive CD4^+^ and CD8^+^ T cell proliferation in peripheral blood during the course of therapy.

**Figure 7:**
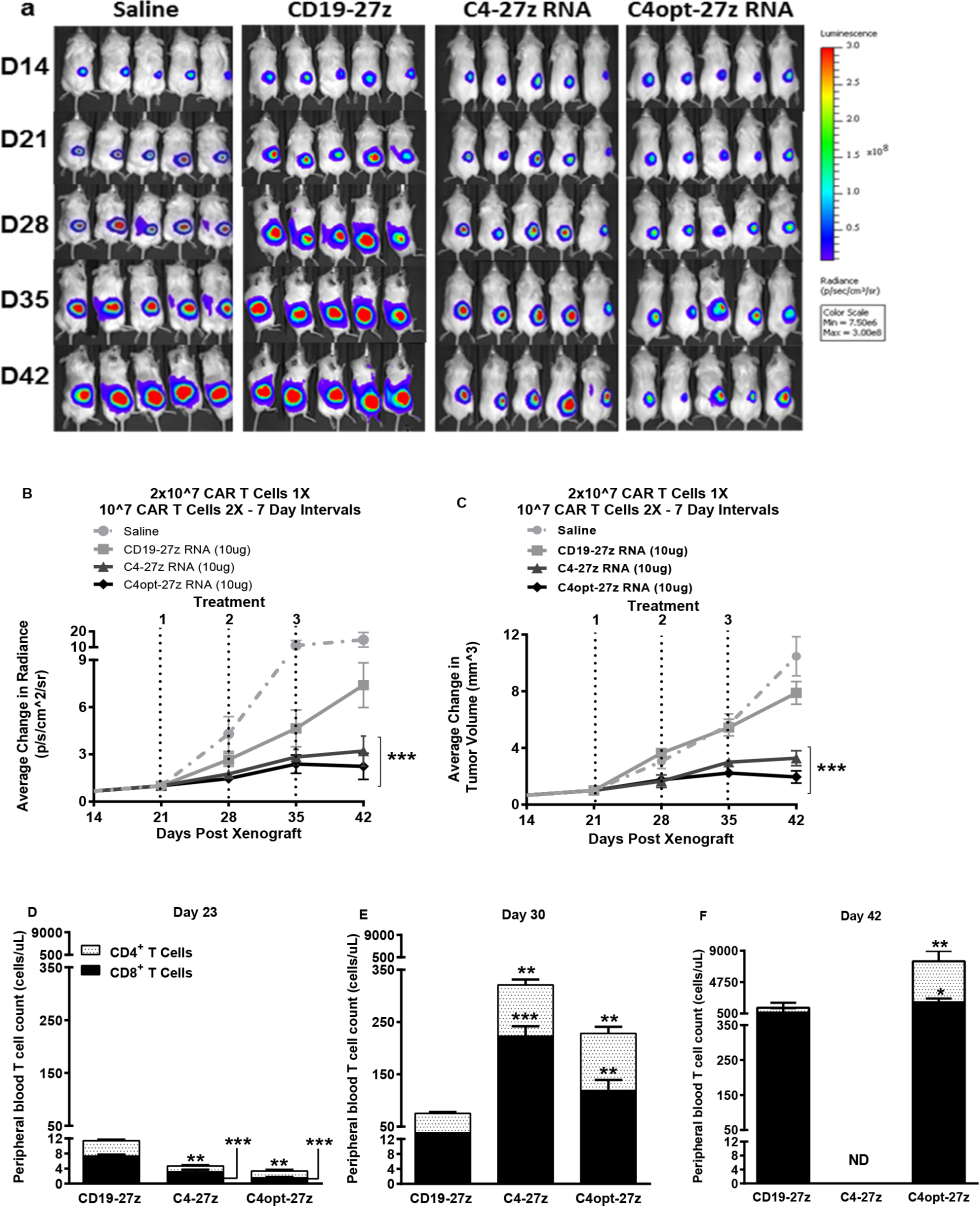
C4-27z and C4opt-27z RNA CAR T cells reduce the progression of solid ovarian cancer *in vivo* **A.** NSG mice bearing solid SKOV3 tumor were treated with on intratumuoral injection of 2 × 10^7^ CAR+ T cells on day 21 (loading dose) followed by a lower maintenance dose of 10^7^ CAR+ T cells on days 28 and 35. **B–C.** Tumor growth was measured weekly by SKOV3 fLUC^+^ bioluminescence (B) and by caliper measurement. C4-27z and C4opt-27z CAR T lymphocytes treated significantly reduced tumor bioluminescence and volume compared to animals receiving no treatment (2-way ANOVA, *p* < .001). **D.** CD4^+^ and CD8^+^ C4-27z and C4opt-27z CAR T cells were initially present at low numbers in peripheral circulation, suggesting FRα-specific CAR T cell migration to specific tumor locales (student *t* test, *p* < .01 - .001). **E.** Repeat administration of C4-27z and C4opt-27z CAR T cells resulted in CD4^+^ and CD8^+^ T cell expansion in peripheral blood during therapy (student *t* test, *p* < .01 - .001) **F.** C4-27z and C4opt-27z CAR T cells are not eliminated after treatment and continue to proliferate despite losing CAR reactivity (student *t* test, *p* < .05 - .01).

Because of the extensive FRα-specific effector functions displayed by C4-27z and C4opt-27z RNA CAR T lymphocytes *in vitro*, we rigorously tested whether these RNA CAR T cells would mediate regression of human cancer *in vivo*. Immunodeficient non-obese diabetic/severe combined immunodeficiency/IL-2Rγc^null^ (NSG) mice were inoculated intraperitoneally (i.p.) or subcutaneously (s.c.) with 5 × 10^6^ fLuc^+^ SKOV3 human ovarian cancer cells. Randomized mice then received one of three treatment regimens depending on the route of inoculation, with i.p. treatments commencing at day 14 and intratumoral treatments on day 21 (see Figure Supplemental 8A–8C for schedules). Regimen one consisted of three i.p. injections of 10^7^ CAR T cells (10–10-10) spaced three days apart in animals inoculated i.p. with SKOV3 cells (Figure 5). In regimen two, i.p. inoculated SKOV3-bearing rodents were administered 2 × 10^7^ CAR-T cells (a loading dose) followed by weekly doses of 10^7^ CAR-T cells for two weeks (maintenance doses; 20–10-10 i.p., Figure 6). A third regimen examined C4opt-27z CAR T cell therapy delivered intratumorally (i.t.) after s.c. tumor inoculation (solid tumor model). Here, mice initially received 2 × 10^7^ CAR-T cells followed by weekly doses of 10^7^ CAR-T cells for two weeks (20–10-10 i.t., Figure 7).

In regimen one, transfer of C4-27z and C4opt-27z RNA CAR T cells by i.p. administration significantly attenuated the progression of disseminated human ovarian cancer which grew rapidly in saline- and CD19-27z CAR-treated mice (Figures 5A–5B, two-way ANOVA, *p* < .001, D28). Similar to *in vitro* findings, C4opt-27z generally outperformed the parental C4-27z RNA CAR T cells in limiting tumor outgrowth. Initially, human CD4^+^ and CD8^+^ T cells in C4-27z and C4opt-27z CAR cohorts were present in lower numbers in the peripheral circulation in comparison to CD19-27z CAR T cells, suggesting early FRα-specific CAR T cell migration to specific tumor sites (Figure 5C, student *t* test, *p* < .01 - .001). Importantly, repeat administration of C4opt-27z CAR T cells resulted in significant expansion of CD4^+^ and CD8^+^ T cells in peripheral blood (Figure 5D, *p* < .001), which correlated with the therapeutic efficacy of the C4opt-27z CAR. Although C4-27z and C4opt-27z CAR T cells were highly beneficial in this paradigm, we hypothesized that the 10–10-10 dosing regimen every third day was suboptimal as tumor growth progressed rapidly once therapy was completed. A similar dosing regimen was shown to be less than ideal in a mouse model of advanced leukemia, as spacing every 3 days did not give sufficient time for individual doses of RNA CAR T cells to complete their effects [19].

Given the drug-like kinetics of RNA CARs [17, 19] and based on our CAR expression data *in vitro* (Figure 1, Supplemental Figure 1), we predicted that a high loading dose (2 × 10^7^) followed by lower maintenance doses (10^7^) at a longer interval of 7 days would result in greater efficacy. Indeed, this second regimen resulted in complete, durable tumor remission in 100% of C4-27z and C4opt-27z RNA CAR T cell treated, tumor-bearing animals (Figures 6A–6B, *p* < .001, D28). Consistent with regimen one, CD4^+^ and CD8^+^ C4opt-27z CAR T cells were first present at low numbers in peripheral circulation (Figure 6C). Likewise, successive administration of C4opt-27z CAR T cells resulted in more extensive CD4^+^ and CD8^+^ T cell expansion in peripheral blood during the course of therapy (Figure 6D).

Encouraged by the therapeutic potential of C4opt-27z CAR T cells using the 20-10-10 weekly dosing regimen, we next tested the efficacy of C4opt-27z RNA CAR T cells against solid tumor, using a s.c. tumor model where RNA CAR therapies, in general, have been only partially effective against different tumor types ([43] and unpublished observations). Importantly, C4-27z and C4opt-27z CAR T cells significantly reduced the rate of solid tumor progression, although the majority of animals did not achieve complete remission (Figures 7A–7C, *p* < .001, D42). Paralleling the results in i.p. animal experiments, CD4^+^ and CD8^+^ C4opt-27z CAR T cells were initially present at low numbers in peripheral circulation compared to control CD19–27z CAR T cells (Figure 7D, *p* < .001) and successive administration of C4opt-27z and C4opt-27z CAR T cells resulted in significant CD4^+^ and CD8^+^ T cell expansion in peripheral blood during the course of therapy (Figure 7E) (*p* < .05 – *p* < .001). Notably, human T cells in the C4-27z and C4opt-27z CAR cohorts continued to persist despite losing CAR reactivity (Figure 7F) (student *t* test, *p* < .05 - .01). Again, the antitumor potency of C4opt-27z was modestly increased relative to the C4-27z CAR, albeit not to a level of statistical significance.

## DISCUSSION

Given the elevated expression of FRα in various human malignancies [32–38], we sought to determine the therapeutic potential of RNA electroporated, FRα-specific C4opt-27z RNA CAR T lymphocytes for the treatment of human cancer. With advances in gene transfer technology, cell cultivation and basic T cell biology, CAR T-cell therapy is becoming more potent, with improved results in clinical trials and increased awareness for the potential of immune toxicity [5–8, 30, 49–53]. Virally transduced CAR T cells possessing one or more costimulatory domains have displayed enhanced antitumor function and persistence in animal models and in the clinic [9–11, 23–27, 29, 30, 54]. However, lentiviral and retroviral CAR T lymphocyte therapies incorporating various costimulatory molecules that were administered to patients have in some cases resulted in amplified cytokine release, ‘septic shock’ and mortality [5, 6, 54–56]. In addition, viral gene transfer poses possible risks associated with stable integration, including the possibility of malignant T cell transformation, though rarely if ever seen, and cumulative off-tumor toxicity due to continued CAR T cell persistence [57, 58]. Conversely, RNA CARs are non-integrating and transiently expressed, and injections of RNA CAR T cells can be quickly discontinued if necessary and toxicity is expected to progressively abate, because, as we show here, CAR T cell surface expression, cytokine release and lytic function gradually decline with time, particularly following CAR activation.

In the limited number of normal tissues in which the α isoform of folate receptor (FR) is expressed, the receptor is restricted to apical (luminal) surfaces, where it is inaccessible via the circulation [39]. Low-level FRα expression is present in the lung, kidney, intestines, heart and choroid plexus, although these folate receptors remain inaccessible to folate or folate-conjugates because of their distinct localization on the apical surfaces of polarized epithelium [39, 59–63]. In healthy patients, folate conjugate uptake has been limited to the kidneys where folate salvage occurs, and renal toxicity has not been observed with folate-chemotherapeutic agents, antibodies or directed T cells [44, 64–66]. However, acute toxicities accompanying administration of anti-folates, including methotrexate, need to be considered, though their mode of toxicity is not well understood. In the current study, assessment of the risk for acute toxicity *in vivo* stemming from C4 FRα-specific RNA CAR T cell therapy is not possible since the C4 CAR recognizes human FRα, but not mouse FRα. However, the C4 CAR has a reduced potential to recognize normal tissue bearing low levels of FRα likely due, in part, to its moderate functional avidity when expressed on the T cell surface following lentivirus transduction [67], suggesting that effective therapy can occur in the absence of significant toxicity. Rapid onset toxicity has been reported elsewhere in a lymphodepleted patient following administration of high numbers of autologous T cells engineered with retrovirus to express HER-2/ErbB2-specific CAR [6]. Despite this, numerous studies of immunogenic folate agents in animals and phase I clinical trials have demonstrated both safety and efficacy of FR targeting [66, 68–70], and an early phase I clinical study using first generation FRα-specific T cells (without a costimulatory domain in the CAR construct) demonstrated that large numbers of these cells can be safety administered to patients without acute toxicity [44]. In another study, intraperitoneal administration of autologous T cells armed with a FRα-specific bispecific antibody to ovarian cancer patients resulted in a 27% intraperitoneal response rate with no on-target toxicities reported [66]. Collectively, these data suggest that a therapeutic window exists wherein effective therapy can be applied in the absence of overt toxicity against normal tissues. However, given that RNA CARs do possess the potential to mediate acute toxicity immediately after infusion; administration of steroids or cyclophosphamide, a T cell lymphodepleting agent [19], may be considered to more rapidly detune or abate FRα-specific RNA CAR T cells *in vivo*.

While unlikely, there exists the potential for FRα-specific RNA CAR T cell toxicity in patients resulting from the need for multiple injections. For example, anaphylaxis as a byproduct of transgene immunogenicity may occur if repeated infusions are given over extended periods of time without lymphodepleting pre-conditioning during or after immunotherapy. In this line, multiple infusions of mesothelin-specific RNA CAR T cells were reported to lead to clinical anaphylaxis in a single patient, although the mechanism was believed to involve IgE antibodies specific to the murine CAR utilized rather than off-tissue effects from targeting FRα mesothelin [71]. Hence, RNA CARs derived from murine antibodies may present safety concerns for use in humans, especially when administered utilizing an extended dosing schedule. To specifically address this, C4opt-27z RNA CAR was fully humanized and infusion schedules were optimized in order to treat over a brief, 3 week period.

To further the potential safety of C4-27z RNA CAR T cell therapy, we codon optimized C4-27z CAR and removed ORFs, creating a novel CAR (C4opt-27z) with reduced likelihood of generating off-target transcription products which could potentially harm individuals or provoke immune rejection of the engineered T cell. Importantly, optimization of C4-27z CAR did not negatively impact antitumor efficacy in any of our experiments. Conversely, when compared to C4-27z CAR, C4opt-27z CAR displays moderately enhanced antitumor activity *in vitro* and enhanced effector function *in vivo*. C4opt-27z also exhibits greater transcriptional efficiency *in vitro*, potentially leading to the production of significantly higher yields of therapeutic RNA at lower cost (Supplemental Figure 9). Hence, C4opt-27z CAR may be superior to C4-27z CAR in terms of clinical application.

RNA CAR methods may permit rapid iterative changes in CAR design and the possibility of moving toward a good manufacturing practice (GMP)-compliant system with potentially lower expenses and less complicated pharmacokinetic testing as opposed to lentiviral or retroviral CARs which can persist for extended periods [17, 19]. Using an RNA approach, we have generated several additional RNA-based CARs at low cost which are currently being screened for potency (Powell and Schutsky, unpublished observations, 2014). As evaluated here, candidate RNA CARs, such as C4opt-27z, demonstrating strong efficacy *in vitro* and in preclinical models may be selected for rapid conversion to viral CARs, as warranted.

Our experiments unequivocally demonstrate that C4opt-27z RNA CAR T lymphocytes have substantial treatment value using a multiple, weighted dosing strategy in aggressive ovarian cancer models. These studies are novel in that they provide the first evidence that FRα-specific, CD27-costimulated, redirected RNA CAR T cells can have potent and enduring antitumor effects *in vivo* without the use of an integrating viral vector system. Immunoreactivity, cytolytic potential and antitumor function of C4opt-27z RNA CAR T cells in our experiments is striking, and begins to rival that of reported virally modified FRα-specific CAR T lymphocytes in other paradigms [28]. However, the benefits of using C4opt-27z RNA CAR T cells against solid tumor, while highly significant, were less dramatic and mimic those reported with HER-2/ErbB2-specific RNA CAR T cells against solid ovarian cancer [43].

In general, FRα-specific RNA CAR T cell therapy is unique because it has the potential to be applied to numerous cancers which express the antigen to differing extents. C4opt-27z redirected T lymphocytes are highly reactive against multiple ovarian cancer lines displaying disparate levels of FRα expression. Our therapy also appears to be highly effective for eradicating other types of cancers which express varying levels of FRα, including breast and pancreatic cancers expressing WT and/or mutant BRCA1 and/or BRCA2 (Schutsky and Powell, 2014, unpublished observations).

Given the temporary, drug-like kinetics of RNA CARs, it not surprising that a loading dose (2 × 10^7^) followed by lower maintenance doses (10^7^ or 5 × 10^6^) at an appropriate half-life interval (7 days) results in improved T cell proliferation and antitumor function as opposed to closer spacing (10^7^ every 3 days), which may not allow for sufficient time for T cells to complete their effects. Others have reported that weekly, weighted dosing as opposed to equal dosing every 3^rd^ day is more effective in treating disseminated leukemia [19]. Although not examined in our experiments, treatment intervals lasting longer than 7 days may allow for xenograft harbor sites to recover and re-seed, making relapse possible [19].

Based on our imaging studies *in vivo*, maximal effects appear to occur within 3–5 days of each administration, with the first administration having the most substantial impact. Notably, RNA CAR T cell expression declines more rapidly *in vivo* than *in vitro*, likely because RNA CAR T cells in immune replete, tumor-bearing mice encounter antigen and homeostatic queues that promote T cell activation, proliferation and RNA metabolism. A total of three doses employed in these studies was a pragmatic decision based on numbers of available T cells using our non-clinical scale manufacturing. It is possible that administering a larger number of T-cell infusions or administering greater numbers of T cells during each infusion might increase the number of animals achieving complete remission, particularly in the solid tumor model which appears more difficult to treat. Conversely, repeat injections of exorbitant numbers of T cells over extended periods could promote the development of cumulative off-target toxicity not otherwise observed under single dosing. Alternatively, a combination of multiple CAR T cell infusions with “erasure” of the prior infused cells using lymphodepletion may allow for optimal efficacy of each cell infusion or reduce the total number of infusions required for complete response, as is the case for particular CARs against disseminated leukemia [19]. Notably, the majority of animals in our models did not succumb to cancer, but rather GVHD, in the very late phase of therapy (∼50 days after first T cell dose) that was likely triggered by xenogeneic responses of human T cells against mouse tissue and the absence of suicide induction systems in NSG animals to effectively eliminate CAR-negative T cells after therapy termination.

Furthermore, it is unclear whether certain costimulatory domains might provide greater benefit in RNA-based CAR T cell therapy. It has been suggested that CAR costimulatory domains carry less influence in RNA models than for viral models, and that cell dose, interval, T lymphocyte age, and lymphodepletion may be critical factors for successful RNA CAR therapy [17, 19]. Still, studies have not rigorously compared RNA CARs containing one or more costimulatory molecules to determine which domains, if any, provide the greatest efficacy. In addition, defining the specific T-cell populations that may be most effectively modified with RNA CARs remains to be determined although our initial results suggest that modifying total CD3^+^ T cells would probably provide more benefit than CD4^+^ T or CD8^+^ T cells alone, especially if utilized for *in vivo* application, based on a greater proinflammatory response and heightened lytic function when both CAR T cell populations are combined. In addition, the T cells utilized for these studies likely contain Tregs, which upon activation with C4opt RNA, could reduce the effectiveness of effector T lymphocytes, as both Tregs and effector T cells would be expected to migrate to FRα-specific tumor locales. Nevertheless, antitumor responses ensued both *in vitro* and *in vivo*.

Despite its strengths, potential limitations of RNA CAR therapy do exist, such as transient CAR expression, the potential need for large *ex vivo* expansion to administer multiple injections, limited proliferation *in vivo* (compared to viral methods) and lack of durable CAR-based antitumor memory cells. In addition, recent safety concerns, including the induction of anaphylactic responses due to the immunogenic nature of multi-dose murine-based CAR regimens need to be addressed [48]. C4-27z CAR constructs are expected to circumvent this issue via incorporation of only human components within the CAR vector. Our experiments also demonstrate that very high amounts of RNA electroporated into T lymphocytes can cause significant antigen-independent activation, including cytokine production and cytolysis. Importantly, such experiments further suggest that appropriate levels of RNA for electroporation can be identified that reduce antigen independent activation effects in order to minimize the potential for adverse events.

In conclusion, RNA CAR T cell therapy, optimized by rigorous preclinical testing as described here, has obvious safety benefits of a non-integrating, transient CAR expression platform and will continue to complement current therapies being developed with retroviral and lentiviral CARs, and, in certain circumstances, may be used as primary therapy. Our results reveal the efficacy of an optimized FRα-specific, CD27 costimulated RNA CAR T cell therapy platform and thus advocate for expedited clinical application of C4opt-27z CAR T cells across the broad range of FRα-expressing cancers.

## MATERIALS AND METHODS

### Construction of *in vitro* transcription (IVT) vectors and RNA electroporation

A lentiviral vector encoding MOv19 anti-FRα scFV coupled with a cytosolic tail comprised of a CD27 costimulatory domain had been previously engineered [28]. A novel, fully human scFV against FRα (C4) was utilized for CAR construction [47]. MOv19 was removed and C4 scFV “swapped” in to create a fully humanized CAR construct, C4-27z (Figure 1A). To generate C4opt-27z, internal ORFs were removed from the C4-27z CAR (Life Technologies, GeneArt, Grand Island, NY) and all codons were optimized, reducing the possibility of undesirable transcription products, increasing translational efficiency and reducing the costs of producing RNA (Supplemental Figures 8A–8B). C4-27z or C4opt-27z CAR constructs were then subcloned into a pD-A.lenti cloning site.2bg.150A vector (PDA) that had been optimized for T cell transfection, CAR expression and RNA production [17, 30]. A similar approach was used to subclone a control CAR, CD19-27z, which does not target FRα^−^ but CD19^+^ cancer cells [17–19].

C4-27z, C4opt-27z and CD19-27z CAR cDNAs were confirmed by direct sequencing and linearized by *SpeI* digestion prior to RNA IVT. The T7 mScript Standard mRNA Production System (Cellscript, Inc., Madison, WI) was utilized to generate capped/tailed IVT RNA. The IVT RNA was purified by phenol-chloroform extraction followed by RNeasy Mini Kit (Qiagen, Inc., Valencia, CA). Purified RNA was eluted in RNase-free water at 1–2 mg/ml and stored at −80°C until use. RNA integrity was confirmed by 260/280 absorbance and visually on an RNA denaturing gel.

### Human T cells

Primary human T cells were isolated from healthy volunteer donors after leukapheresis and purchased from the Human Immunology Core at the University of Pennsylvania. All specimens were collected under a protocol approved by a University Institutional Review Board, and written informed consent was obtained from each donor. T cells were cultured in complete media (RMPI 1640 supplemented with 10% heat-inactivated FBS, 100 U/mL penicillin, 100 ug/mL streptomycin sulfate, 10 mM HEPES) and stimulated with anti-CD3 and anti-CD28 mAbs-coated beads (Invitrogen) as described [72]. Human recombinant interleukin-2 (IL-2; Novartis) was added one day after CD3/CD8 stimulation, then every other day at 50–100 IU/mL final concentration, for 14 days, until T cell number reached approximately 5 × 10^8^ cells. At approximately 14 days, T cells became “rested down,” as determined by both decreased growth kinetics and cell sizing using the Multisizer 3 Coulter Counter (Beckman Coulter). T cells were washed twice with Opti-Mem and suspended in Opti-Mem at a final concentration of 10^8^ / mL prior to electroporation. Subsequently, T cells were mixed with 10 μg IVT RNA/0.1 mL T cells and electroporated in a 2-mm cuvette (Biorad) using an ECM830 Electro Square Wave Porator (Harvard Apparatus, BTX, Hollison, MA) at 500V, 700usec, 1 pulse. Viability post transfection ranged from 65–85%. Viable T cells used for experiments had 95–100% CAR expression at the time of use, except in experiments where time-dependent effects were assessed.

### Cell lines

Cell lines used in immune based assays include the established human ovarian cancer cell lines SKOV3, A1847, OVCAR3, A2780 and C30. For cell lysis assays, target cancer cell lines were transduced to express firefly luciferase (fLuc^+^). For specificity controls, the mouse malignant mesothelioma cell line, AE17 (provided by Steven Albelda, University of Pennsylvania), was transduced with lentivirus to express FRα (AE17.FRα). K562 and CD19-expressing K562 (K562.CD19), human erythroleukemic control cell lines, were provided by Michael Milone of the University of Pennsylvania. All tumor lines were maintained in RPMI-1640 (Invitrogen) supplemented with 10% (v/v) heat-inactivated FBS, 2 mM L-glutamine, 100 μg/mL penicillin and 100U/mL streptomycin. Cell lines were routinely tested for mycoplasma contamination.

### Flow cytometric analysis

The following mAbs were used for phenotypic analysis: i. biotin-SP-conjugated AffiniPure rabbit anti-human IgG (H+L) and ii. biotin-SP-conjugated AffiniPure rabbit anti-mouse IgG (H+L) (Jackson, West Grove, PA); iii. biotin-conjugated recombinant human FOLR1 (RD Systems, Minneapolis, MN / Thermo Scientific, Rockford, IL); i.v. streptavidin-APC (BD, San Jose, CA); v. BD ViaProbe (7-AAD); vii. anti-human CD3-FITC, anti-human CD4-FITC, anti-human CD8-FITC (eBioscience). In adoptive immunotherapy experiments, T cells from peripheral blood were obtained via retro-orbital bleeding and stained for the presence of human CD45, CD3 and CD8 using anti-human CD45-PE, anti-human CD3-PerCP/Cy5.5 and anti-human CD8-APC (Biolegend, San Diego, CA). After gating on the human CD45^+^ population, the CD3^+^ and CD8^+^ subsets were quantified using TruCount tubes (BD Biosciences) with known numbers of fluorescent beads as described in the manufacturer's instructions. Tumor cell surface expression of FR was detected by Mov18/ZEL antibody (Enzo Life Sciences). Flow samples were run using BD FACS Canto and cytometric data analyzed by FlowJo 7.6.5 software.

### Cytokine release assays

Cytokine release assays were performed by co-culture of 10^5^ T cells with 10^5^ target cells per well in 96-well round bottom plates in a final volume of 200 ul of RPMI complete media. After ∼24 hrs, supernatants were assayed for presence of IFN-γ and IL-2 using ELISAs, according to manufacturer's instructions (Biolegend). IFN-γ, IL-2, IL-4, IL-10, MIP-1A and TNF-α cytokines were measured by flow cytometry with Cytometric Bead Array (BD Biosciences), based on protocols from the manufacturer. All cytokine data are represented as a mean of triplicate wells +/− SEM.

### Cytotoxicity assays

For cell-based bioluminescence assays, 3 × 10^4^ firefly Luciferase expressing (fLuc+) tumor cells were cultured in complete RMPI media in the presence of different ratios of RNA transfected CAR T cells in a 96-well Microplate (BD Biosciences). After incubation for ∼ 20 hr at 37°C, 100 μl media was removed from each well and bioluminescence was assessed using the Tropix Luc-Screen Assay (Applied Biosystems, Bedford, MA), according to manufacturer's instructions. Percent tumor cell viability was calculated as the mean luminescence of the experimental sample divided by the mean luminescence of the input number of target cells utilized in the assay times 100. All cytolytic data are represented as a mean of six wells +/− SEM.

### Xenograft models of ovarian cancer

Animals were obtained from the Stem Cell and Xenograft Core of the Abramson Cancer Center, University of Pennsylvania. 12- to 20-week-old nonobese diabetic/severe combined immunodeficient/γ-chain^−/−^ (NSG) mice were bred, treated, and maintained under pathogen-free conditions in-house under University of Pennsylvania Institutional Animal Care and Use Committee–approved protocols. NSG mice were inoculated either intraperitoneally (i.p.) or subcutaneously (s.c.) with 5 × 10^6^ SKOV3 fLuc^+^ cells in the abdomen or flank on day 0. For each experiment, five mice were randomized per group before treatment. In both i.p. models, tumors were widely disseminated at 14 days, as measured by bioluminescence imaging. In the s.c. model, tumors were detectable by imaging at 14 days, becoming physically palpable by 21 days. 13 or 20 days after tumor inoculation, human primary T leukocytes were electroporated with C4-27z, C4opt-27z or CD19-27z CAR RNA, then recovered in RPMI media containing with 50–100 IU/ml IL-2 until injection the following day. In the initial i.p. model, mice were injected with 10^7^ CAR-T cells every 3 days for 9 days, beginning at day 14 (10-10-10 i.p.). In subsequent experiments, rodents were administered 2 × 10^7^ CAR-T cells on day 14 followed by one weekly dose of 10^7^ CAR-T cells for two weeks (20–10-10 i.p.). The solid tumor model was similar to the second i.p. model, except that tumor inoculation was subcutaneous and treatment was delivered intratumorally (i.t.). Additional control groups of i.p. and i.t. animals received either saline injections or T cells containing no RNA (see Supplemental Figures 9A–9C for schedules). Each *in vivo* experiment was repeated at least twice with similar results. Tumor dimensions were measured with calipers (s.c.) and tumor volumes calculated with the following formula: *V* = 1/2(length × width^2^), where length is greatest longitudinal diameter and width is greatest transverse diameter. Animals were imaged before T-cell transfer and every week thereafter.

### Bioluminescence imaging

Imaging of tumor was performed with the Xenogen IVIS imaging system and the photons emitted from fLuc^+^ cells within the animal body were quantified with the Living Image Version 3.0 software (Xenogen). In brief, mice bearing fLuc^+^ SKOV3 tumor cells were injected intraperitoneally with D-luciferin (150 mg/kg stock, 100 μl of D-luciferin per 10 g of mouse body weight) suspended in PBS, and imaged under isoflurane anesthesia after ∼ 10 minutes. Pseudocolor images representing light intensity (blue, least intense; red, most intense) were generated with Living Image. Imaging findings were confirmed at necropsy.

### Statistical analysis

The data are reported as means +/− SEM. Statistical analysis was performed by the use of 2-way ANOVA for the tumor burden (tumor volume, photon counts). Student *t* test was used to evaluate differences in CAR expression or T cell number in peripheral blood. GraphPad Prism 5.0 (GraphPad Software) was utilized for the statistical calculations. *P* < .05 was considered significant.

## SUPPLEMENTARY FIGURES


