# Supplementary material for: Rigorous optimization and validation of potent RNA CAR T cell therapy for the treatment of common epithelial cancers expressing folate receptor
Source: Oncotarget. 2015 Sep 2;6(30):28911–28. doi: 10.18632/oncotarget.5029 (PMC4745700; doi:10.18632/oncotarget.5029)
Supplement: Supplementary file 1 [file oncotarget-06-28911-s001.pdf]

SUPPLEMENTARY FIGURES

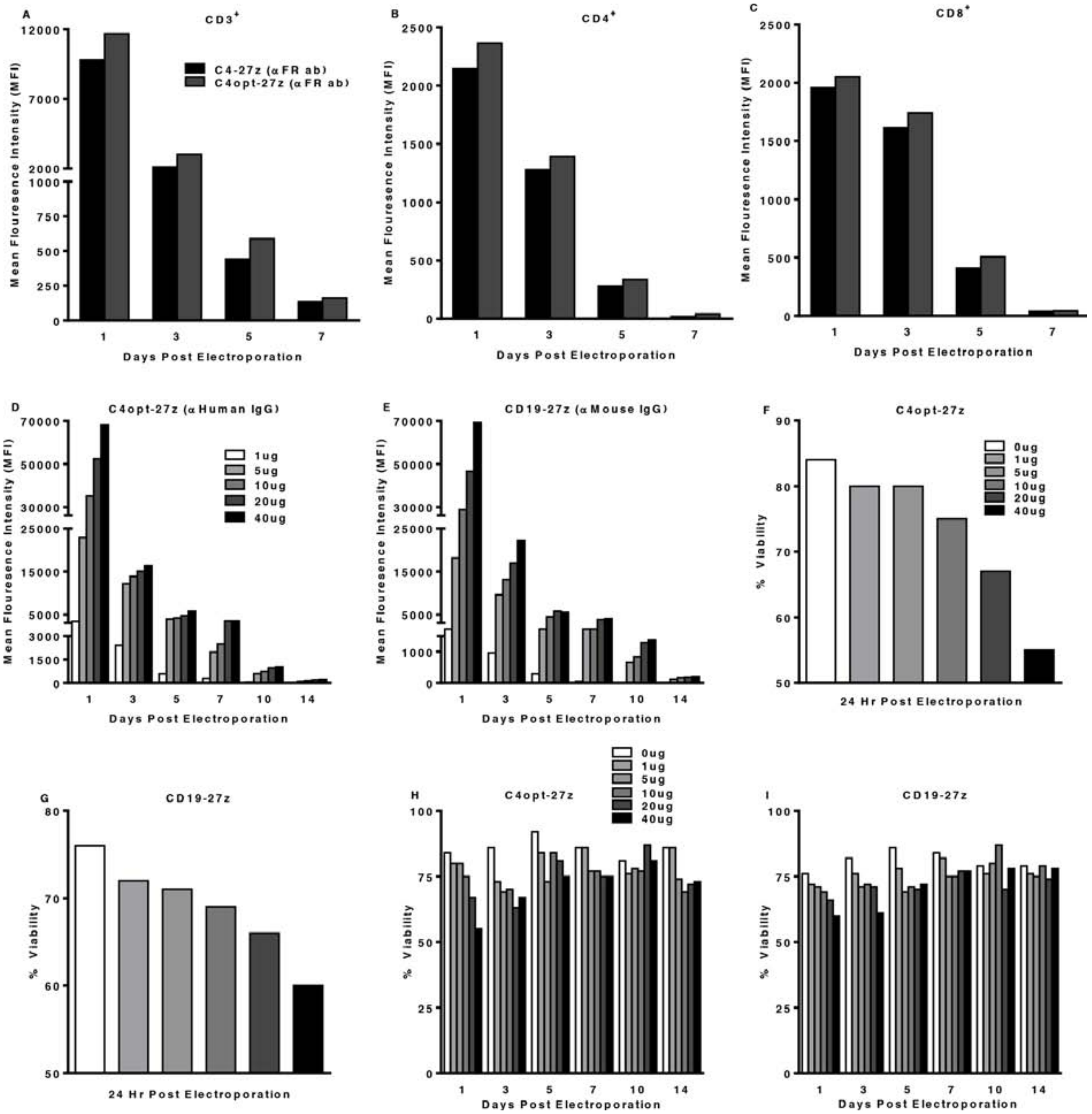

Supplementary Figure S1: C4 CAR expression is FRα-specific and time-dependent / Expression and viability of CAR-transfected human T cells depend on the concentration of input RNA. A–C. CAR expression as measured by mean fluorescence intensity (MFI) at different times after electroporation with 10 µg C4-27z or C4opt-27z. Specificity for FRα-specific CARs was confirmed in different T cell populations using biotinylated, recombinant human α-folate protein. **D–E.** CAR expression is dependent on the concentration of RNA used for electroporation. **F–G.** Viability (24–72 hr) is inversely proportional to RNA concentration. **H–I** High concentrations of CAR RNA do not produce irreversible toxicity in T cells as viable cells replace those damaged by electroporation.

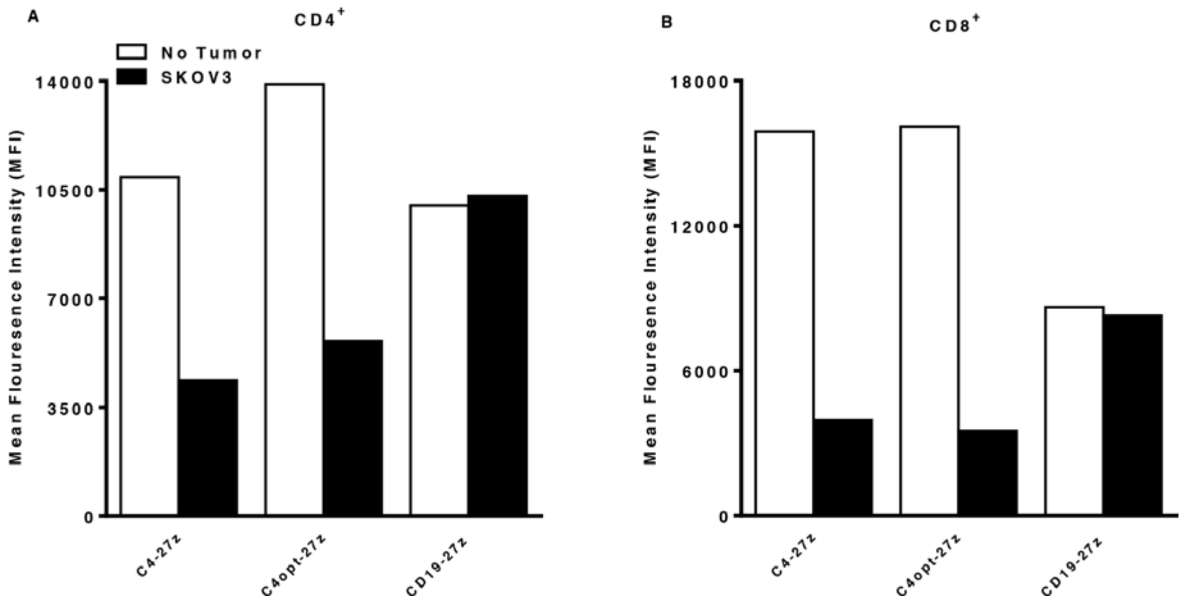

Supplementary Figure S2: C4-27z and C4opt-27z CAR T cell expression rapidly declines after FRα-specific antigen encounter in both CD4$^+$ and CD8$^+$ T cell populations. Expression of C4-27z and C4opt-27z, but not CD19-27z (control CAR) decline rapidly (measured at 72 hr) after 24 hr co-incubation with FRα$^+$ SKOV3 tumor on day 1.

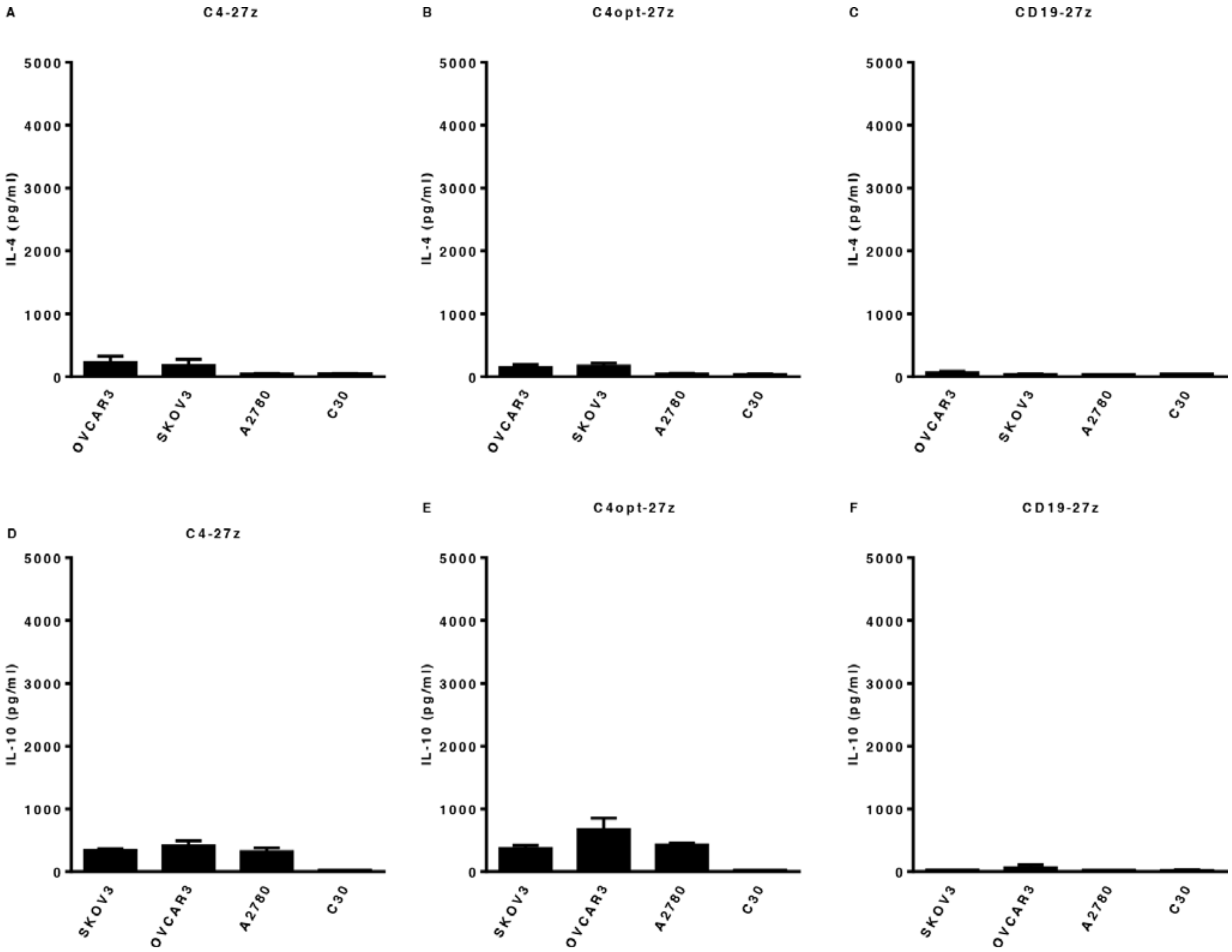

Supplementary Figure S3: Limited Th-2 cytokines are secreted by electroporated C4-27z and C4opt-27z RNA CAR T cells in response to FRα$^+$ tumor cells. A–B, D–E. Antigen-specific IL-4 & IL-10 production by FRα-specific RNA CAR-transfected T cells ~ 24 hr after coculture with the indicated tumor lines at a 1:1 ratio, measured with cytokine bead array. **C, F.** CD19-27z CAR-T cells were used as negative controls. Results are expressed as a mean +/− SEM of triplicate wells from 1 of at least 2 separate experiments.

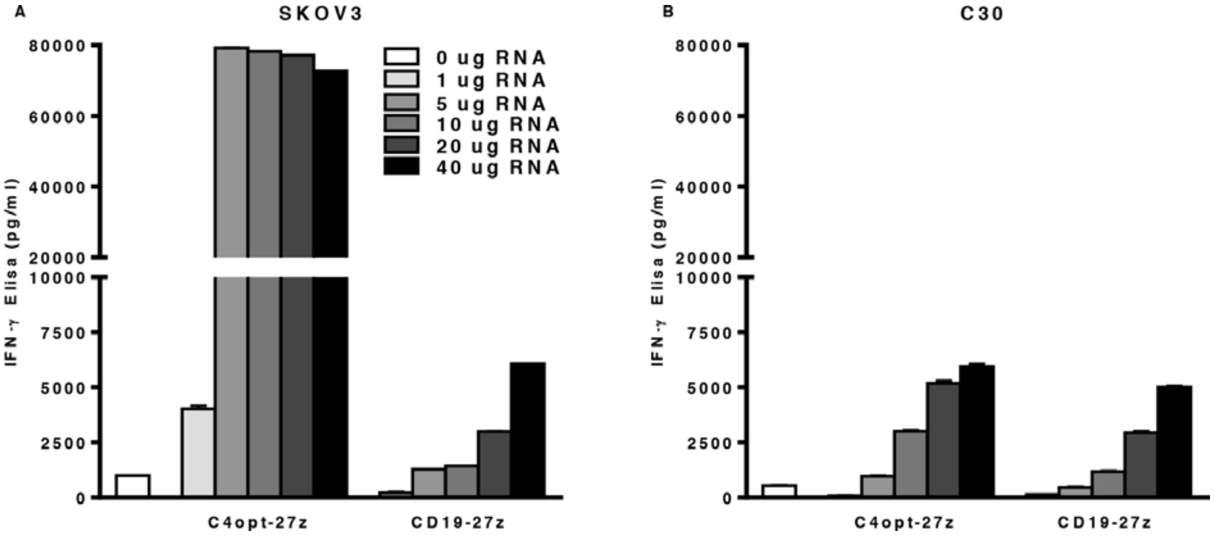

Supplementary Figure S4: Antigen-specific (FRα) and non-specific CAR T cell reactivity depends on the concentration of input RNA. A. Levels of IFN-γsecreted by C4opt-27z and CD19-27z CAR T cells after coculture with FRα^{+} SKOV3 and electroporated with successively higher RNA concentrations. Both FRα-specific cytokine secretion by C4opt-27z CAR T cells and non-specific production by CD19-27z CAR T cells increased based on the concentration of input RNA. **B.** C4opt-27z and CD19-27z CAR T cells were electroporated with escalating doses of RNA and cocultured overnight with FRα^{-} C30 tumor. High RNA concentrations resulted in significant off-target CAR activation, suggesting concentrations below 10ug may minimize such effects.

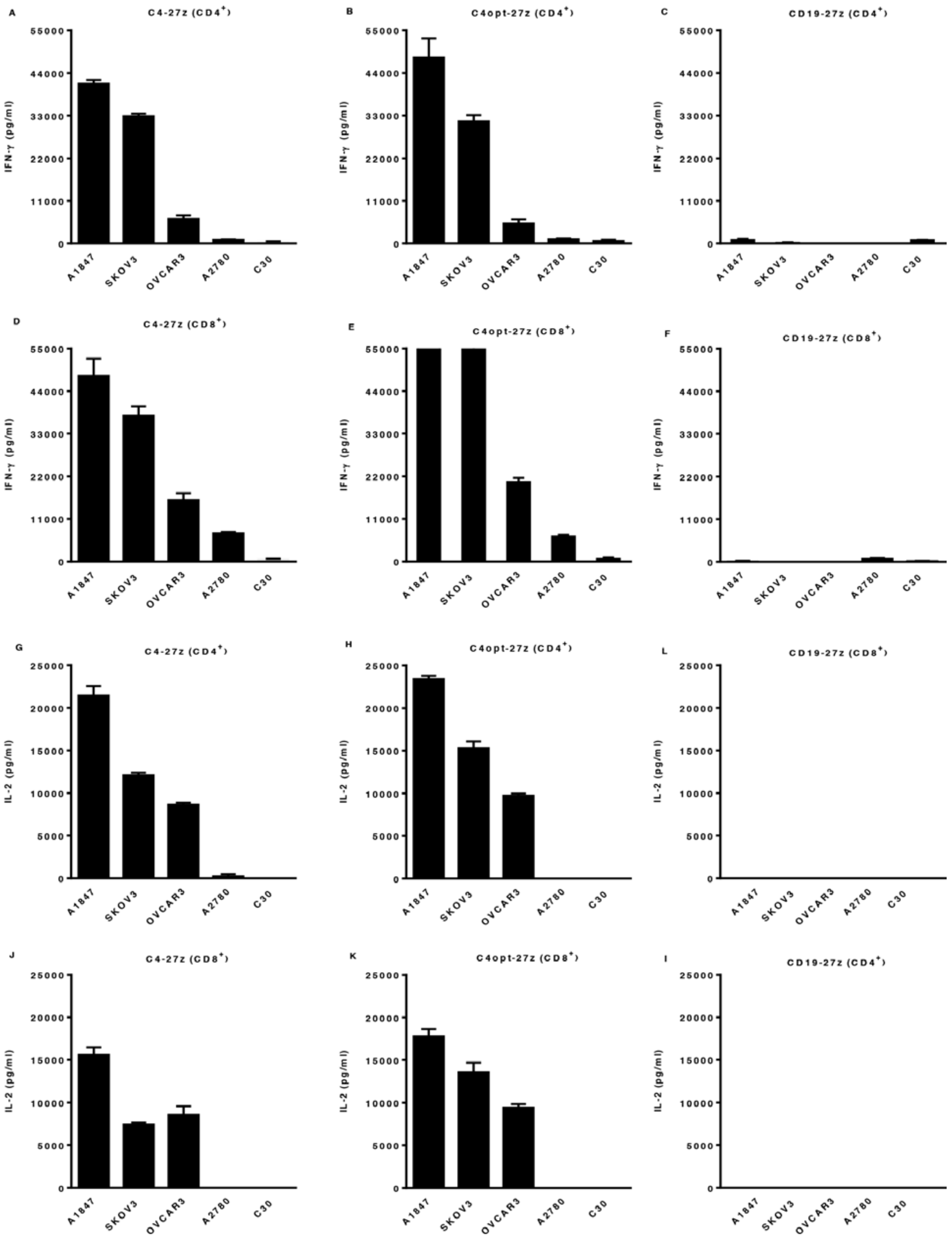

Supplementary Figure S5: Th-1 cytokines are secreted by both CD4$^+$ and CD8$^+$ C4-27z and C4opt-27z RNA CAR T cells in response to FRα$^+$ tumor cells. A–B, D–E, G–H, J–K. Antigen-specific IFN-γ & IL-2 cytokine production by FRα-specific C4-27z and C4opt-27z RNA CAR-transfected T cells ~ 24 hr after coculture with the indicated tumor lines at a 1:1 ratio, measured with Elisa. **C, F, I, L.** CD19-27z CAR-T cells were used as negative controls. Results are expressed as a mean +/− SEM of triplicate wells from a single donor.

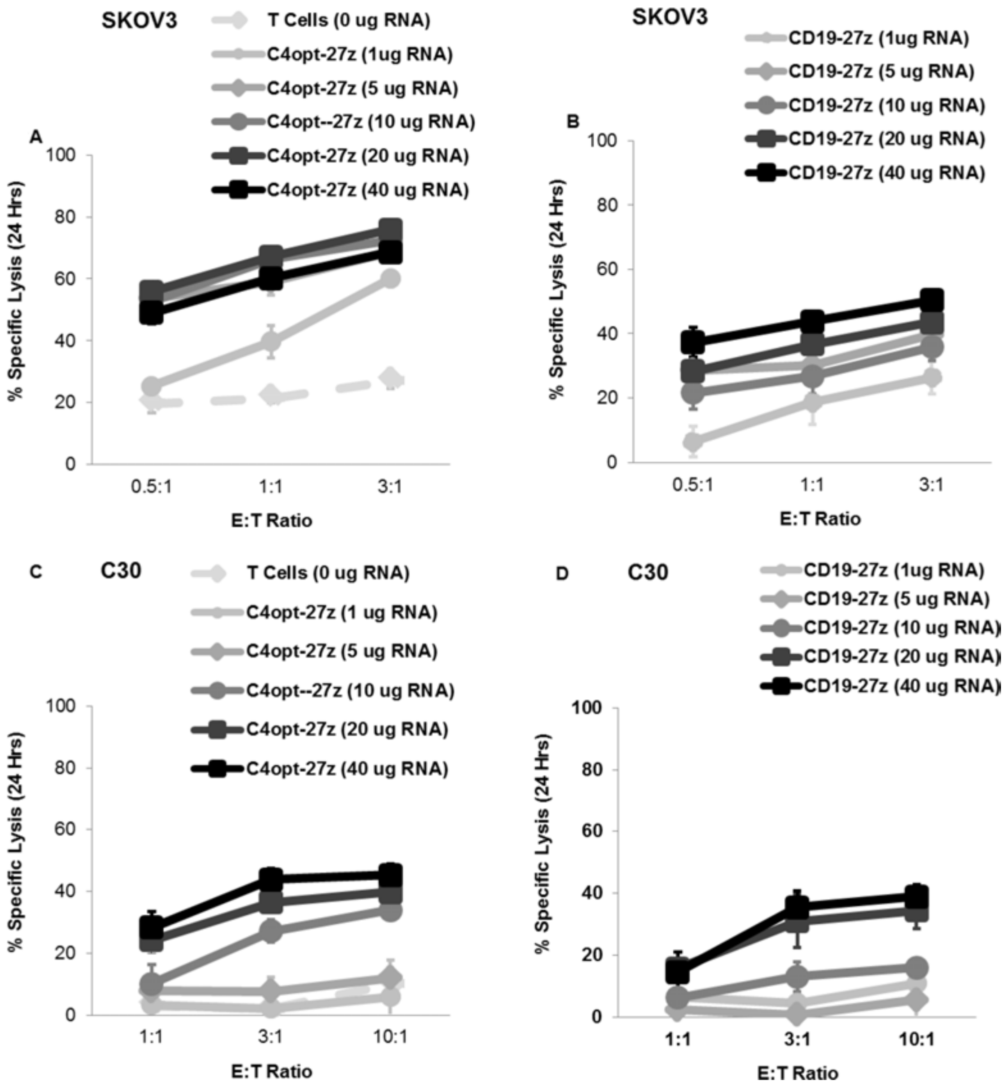

Supplementary Figure S6: FR-specific and non-specific CAR T cell killing depends on the concentration of input RNA. A–B FRα-specific lytic function of C4opt-27z CAR T lymphocytes and unrelated lysis by CD19-27z CAR T cells was determined using successively higher RNA concentrations. Both FRα-specific killing by C4opt-27z and non-specific killing by CD19-27z CAR T cells was dependent on the concentration of input RNA. **C–D.** Cytolysis of C4opt-27z and CD19-27z CAR T cells that were electroporated with escalating doses of RNA was measured after coculture overnight with FRα^- C30 tumor. Higher RNA concentrations resulted in significant off-target lysis, suggesting lower RNA concentrations may minimize such effects.

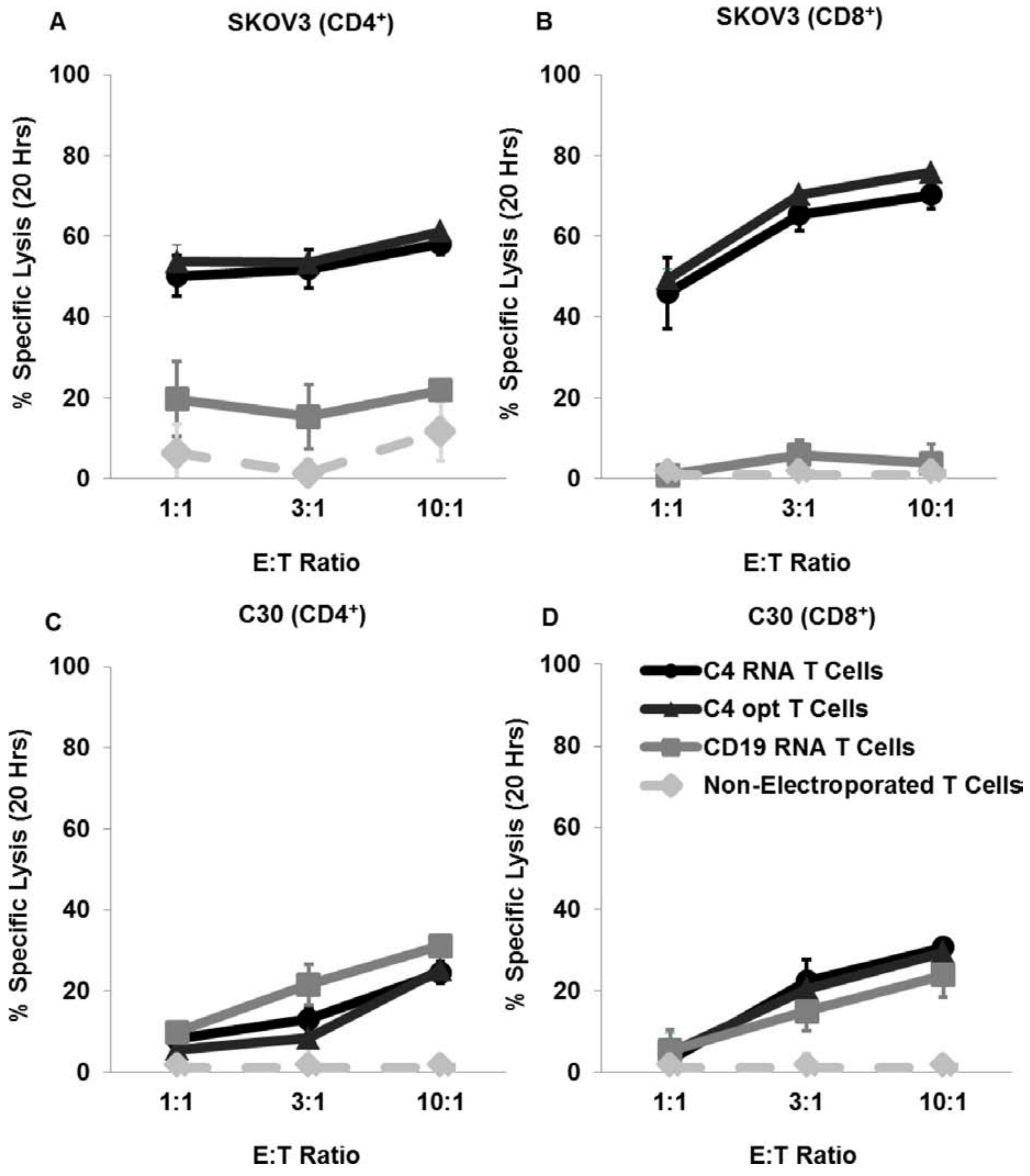

Supplementary Figure S7: CD4+ and CD8+ CAR-transfected, FRα-specific T cells show differing lytic function in a bioluminescent killing assay. A–B. CD4+ and CD8+ electroporated C4-27z and C4opt-27z CAR T cells display FRα-specific lytic function, though to differing extents, and similar levels of non-specific lysis **C–D**.

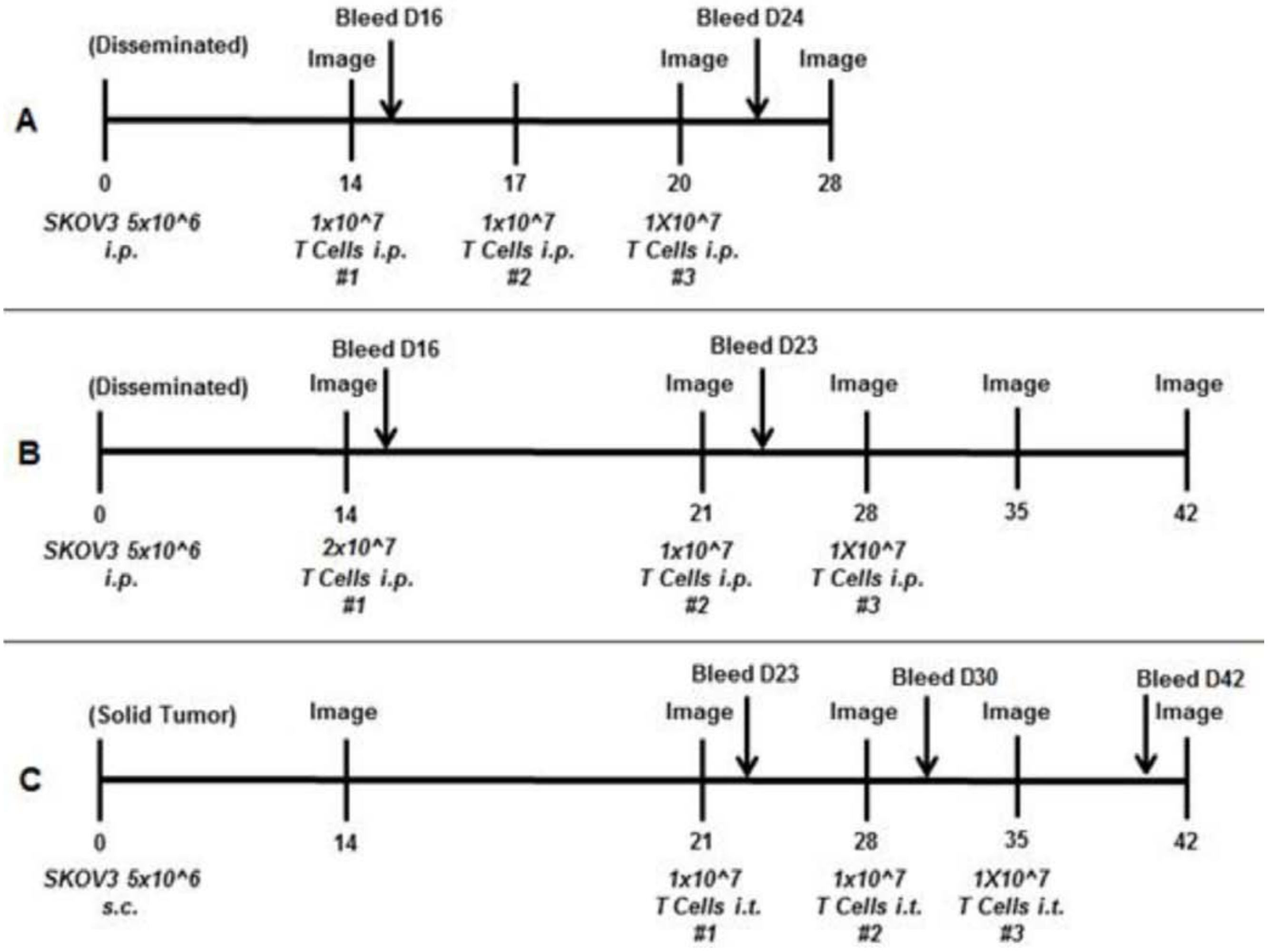

Supplementary Figure S8: Dosing Schedules. A. $10^7 \times 10^7 \times 10^7$ CAR T cells every 3 days intraperitoneal tumor / treatment model. **B.** $2 \times 10^7 \times 10^7 \times 10^7$ weekly intraperitoneal tumor / treatment model. **C.** $2 \times 10^7 \times 10^7 \times 10^7$ weekly solid tumor / intratumoral treatment model.

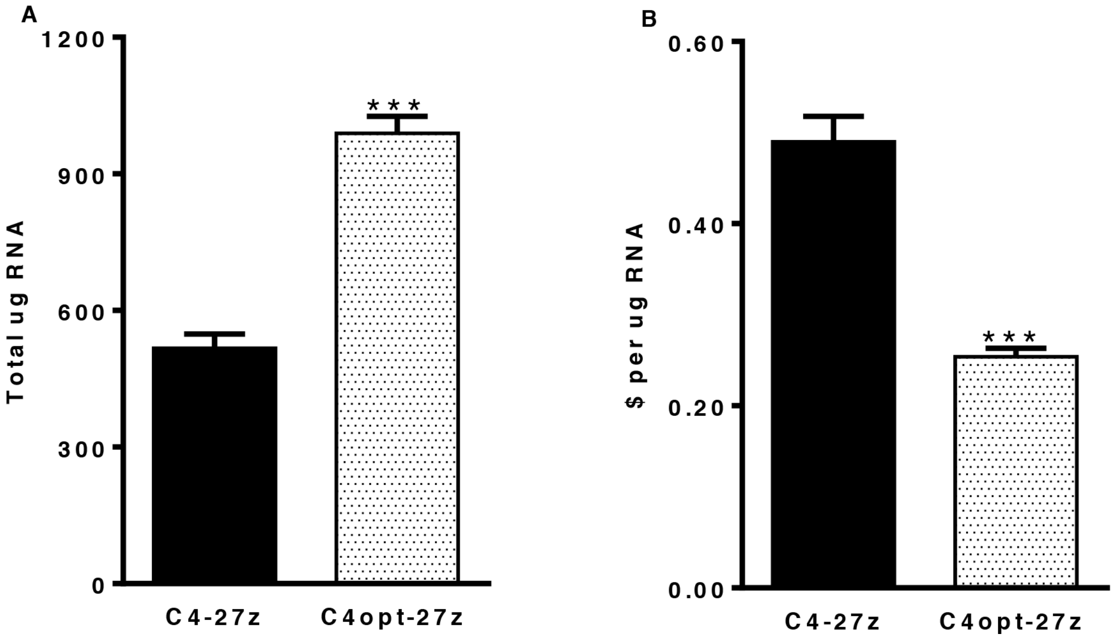

Supplementary Figure S9: Optimization of C4-27z CAR results in greater RNA production at lower cost. Removal of open-reading frames (ORFs) and codon optimization of C4-27z CAR resulted in the creation of C4opt-27z CAR, a higher yield **A.** and lower cost **B.** DNA construct.
